# A review of FDA approved drugs and their formulations for the treatment of breast cancer

**DOI:** 10.3389/fphar.2023.1184472

**Published:** 2023-07-28

**Authors:** Mohini Chaurasia, Romi Singh, Srija Sur, S. J. S. Flora

**Affiliations:** Era College of Pharmacy, Era University, Lucknow, Uttar Pradesh, India

**Keywords:** breast cancer, FDA, formulations, nanoformulation, nanoemulsion, liposomes, cytotoxic drugs, hormonal and targeted drugs

## Abstract

Breast cancer is one of the most diagnosed solid cancers globally. Extensive research has been going on for decades to meet the challenges of treating solid tumors with selective compounds. This article aims to summarize the therapeutic agents which are either being used or are currently under approval for use in the treatment or mitigation of breast cancer by the US FDA, to date. A structured search of bibliographic databases for previously published peer-reviewed research papers on registered molecules was explored and data was sorted in terms of various categories of drugs used in first line/adjuvant therapy for different stages of breast cancer. We included more than 300 peer-reviewed papers, including both research and reviews articles, in order to provide readers an useful comprehensive information. A list of 39 drugs are discussed along with their current status, dose protocols, mechanism of action, pharmacokinetics, possible side effects, and marketed formulations. Another interesting aspect of the article included focusing on novel formulations of these drugs which are currently in clinical trials or in the process of approval. This exhaustive review thus shall be a one-stop solution for researchers who are working in the areas of formulation development for these drugs.

## 1 Introduction

Among all the listed causes of mortalities, Cancer remains one of the leading causes of human death throughout the world every year. As reported by WHO, nearly 10 million deaths have been accounted for Cancer in the year 2020 wherein 2.26 million cases have been reported for Breast Cancer surpassing other leading solid cancers like Lung cancer, Colon and Rectal cancer as well as Prostate cancer.

Breast cancer is the most prevalent cancer among females worldwide. WHO Global Breast Cancer Initiative (GBCI) endeavors to reduce global breast cancer mortality by 2.5% per year thus avoiding 2.5 million breast cancer deaths between 2020 and 2040. The incidence of breast cancer has been rising globally, over the years. According to 2021 estimates breast cancer accounted for about 30% of all the new cancers diagnosed in women in the United States with approx. 15% leading to death. This statistic emphasizes that current research on breast cancer is of utmost importance. Reportedly, incidences of breast cancer have increased to an extent that one new breast cancer is diagnosed every 18 s. Though the mortality rate of breast cancer has changed a little over the years, the survival rate, however, has also increased due to awareness campaigns, early detection programs, and continuous research to develop new drug molecules or new formulations for the treatment of the disease (Roy et al., 2022). Solid Cancers are characterized by the growth of abnormal tissue mass either as sarcomas or carcinomas. Whereas sarcomas arise from the embryonic mesoderm; carcinomas develop from the epithelium or the upper lining of the internal organs. Cancers originating mostly in the breast, stomach and lungs, are carcinomas.

The Breast is made up of glandular tissues which produce and ensure the passage of breast milk, stromal tissues made up of fatty and fibrous connective tissues acting as supporting tissues and lymphatic tissues connected to immune system which draws out the cellular fluids in the form of waste materials. Breast cancer can be classified in different types according to the sites and invasiveness. Most of the tumors in different parts of the breast develop as benign fibrocysts that becomes malignant when it starts to invade different tissues and spreads to the other organs. Breast cancer is characterized by the over-expression of hormone-specific receptors or epidermal growth factor receptors. Further, the classification of Breast cancer includes the consideration of the stage and grade of Breast cancer (Najafi et al., 2021). Considering the amount of ongoing research, there are a variety of drugs and treatments either available or coming into existence. Treatments including chemotherapy and personalized therapy take the help of established drugs including aromatase inhibitors (Miller, 2003), receptor modulators or degraders. The purpose of this article is to review the available information on all FDA-approved drugs for breast cancer with respect to chemical information, drug class, treatments, clinical pharmacology, dosage form, mechanism of action, clinical efficacy for breast cancer and the future direction of ongoing research. An effort has also been made in the review article wherein all registered drug molecules are presented and discussed. We believe that this exhaustive review will become the single stop for reference on all perspectives of an anticancer drug molecule that are present in the market and approved by the FDA to date for breast cancer management.

## 2 Breast cancer statistics

Cancer survival is typically described in terms of relative survival, which is a measure of life expectancy among cancer patients compared to that among the general population of the same age, race, and sex. The 5-year relative survival rate for all cancers combined has increased substantially since the early 1960s, from 39% to 68% among white people and from 27% to 63% among black people (U.S. Cancer Statistics Data, 2021). American Cancer Society (ACS) estimates the count of new cancer cases as well as deaths in the United States every year. The United States is expected to witness 1, 918, 030 new cancer cases and 6, 09, 360 cancer deaths in the year 2022-2023. As per the predicted statistical analysis of the data obtained, it has been concluded that the progress for disease occurrence and recovery has been stagnant for Prostate cancer in men and Breast cancer in females (Siegel et al., 2022).

The commonest malignancy among women globally is breast cancer and has even surpassed the lung cancer incidences which was supposed to be the most common cancer globally in 2020. Epidemiology estimates indicate that breast cancer will have a global burden of almost 2 million by the end of 2030 (Switon and Hill, 1977). In 2020, 2.3 million women were diagnosed with breast cancer accounting for about 6, 85, 000 deaths, globally (WHO, 2021). The statistics are alarming as it became even worse in 2022. In 2022–2023, the United States will witness invasive breast cancer that will be newly diagnosed in women and men to an estimated count of 287, 850 and 2, 710 respectively. Additionally, 51, 400 cases of ductal carcinoma *in situ* (DCIS) will be diagnosed in women. An estimated 43, 780 breast cancer deaths (i.e., 43, 250 in women, 530 in men) are likely to occur in 2022.

## 3 Breast cancer and treatment methodologies

Breast cancer may be classified as Ductal BC, Lobular BC or Connective tissue BC, depending on the type of cell involved. Some cancer cells have overexpressed receptors for few hormones, i.e., Estrogen, Progesteron or HER-2 gene (Orrantia-Borunda et al., 2022) whereas, others may have other molecular markers miRNAs (let-7, miR-155, miR-150, miR-153) and mutations (p53, BRCA 1 and 2 genes). This knowledge help doctors to design more personalized, targeted and effective therapy for the patient (Curigliano and Criscitiello, 2014; Nounou et al., 2015).

**FIGURE 1:**
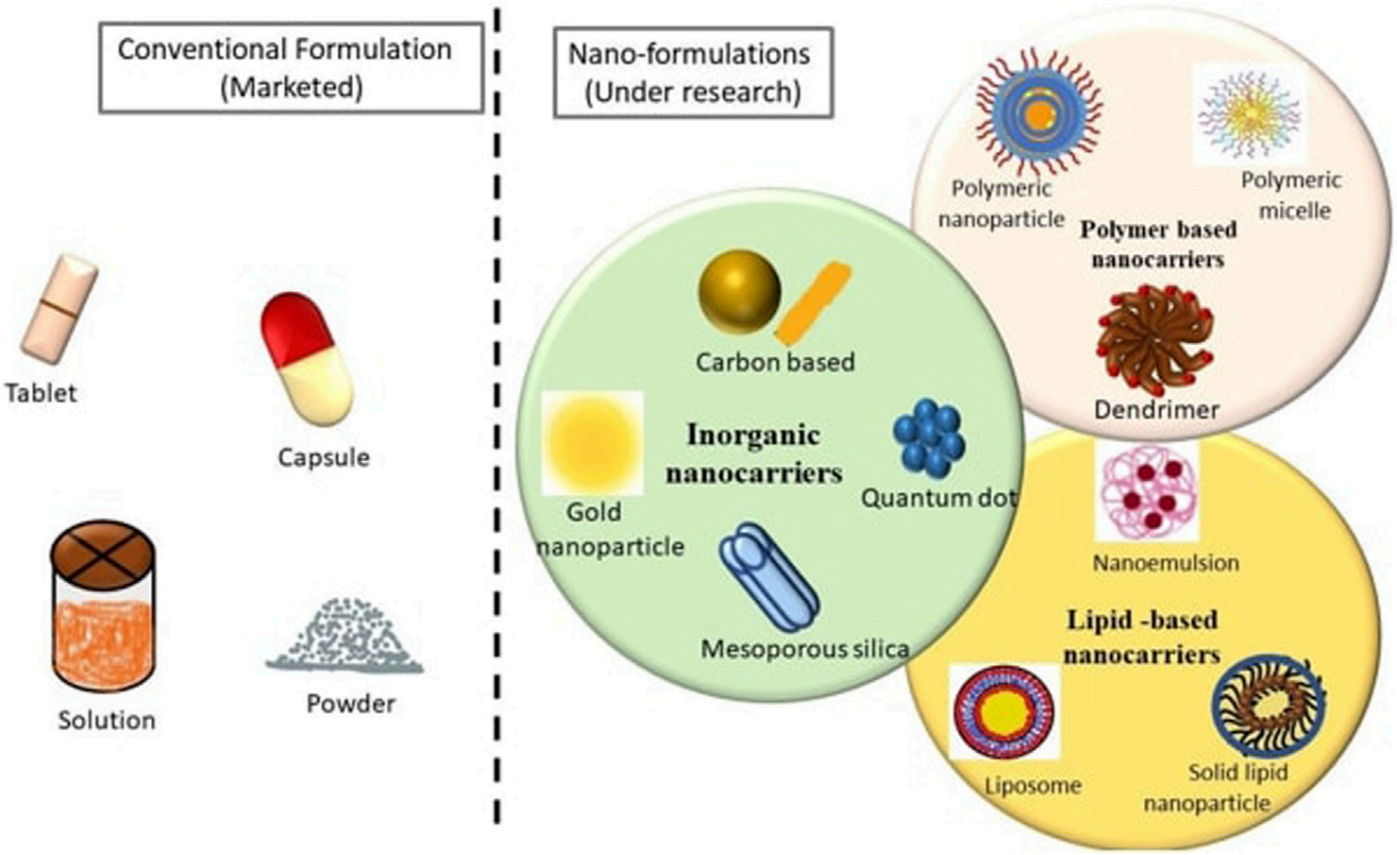
Pictorial representation of Conventional and Novel formulations of various FDA approved anticancer Drugs used for Breast Cancer Indications.

Breast Cancer is highly curable if it is detected at an early stage. The choice of medication varies with menopausal status. The nonmetastatic breast cancer has three treatment phases. The preoperative phase uses systemic endocrine or immunotherapies (ER, PR or ERBB2 positive cases). Preoperative chemotherapy may also be used and is the only option when tumors have none of the three receptors. The options for the surgical phase with similar survival rates includes; a lumpectomy with radiation or a mastectomy (Figure 1). The postoperative phase includes radiation, endocrine therapy, immunotherapy, and chemotherapy (Bequet-Romero, 2018; Bhushan et al., 2021; Trayes and Cokenakes, 2021; Cancer, 2023). The metastatic breast cancer cannot be cured but can be treated for longer life expectancy and quality of life. The biggest disadvantage of conventional chemotherapy is its unspecific toxicity, undesirable side effects, and low therapeutic indices with the development of drug resistance (Hart et al., 2021). Chemotherapy uses anti-cancer drugs that can be administered either orally or intravenously. The main function of the drug is to travel through the bloodstream to reach the cancer cells. Adjuvant chemotherapy is considered to ensure the killing of any cancer cell left or prevent the spread of remaining cells after surgery. Whereas, on the other hand, neoadjuvant therapy is to be applied to shrink the tumor to avoid less extensive surgery (Kerr et al., 2022). If after neoadjuvant chemotherapy, malignant tumor cells are still noticed after surgical removal of the cancer cells, more chemotherapy is needed to be offered to reduce cancer recurrence (Orrantia-Borunda et al., 2022).

Cancer chemotherapeutic drug delivery through the oral route has always been challenging due to first-pass metabolism, gastrointestinal side effects, and low bioavailability, Conversely, however, the oral route is the most accepted route of administration due to patient compliance. There are wide varieties of dosage forms, drug carriers, and drug delivery systems for anticancer drugs, ranging from the conventional mode of treatment to novel drug delivery systems, arising as promising alternative options for breast cancer management.

**FIGURE 2:**
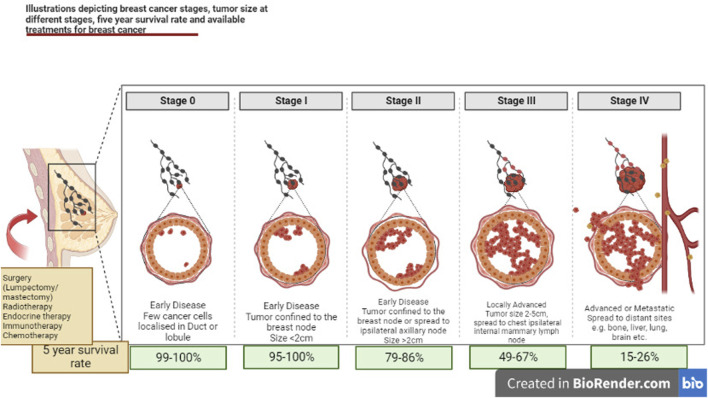
Illustrations depicting mechanism for various stages of breast cancer, tumor size at different stages, 5-year survival rate and available treatments for breast cancer.

The common dosage forms available in the market for oral administration include tablets, capsules, suspensions and others. Even when the medicines are not given through the oral route and given through the parenteral route, the formulations which are mostly available in the market are the ones that are considered conventional dosage forms such as solutions, dispersible powders and so on (Nounou et al., 2015). Tremendous research is going on for nanotechnology-based therapeutics in order to overcome the shortcomings of conventional dosage forms (Hartshorn et al., 2019; Orrantia-Borunda et al., 2022; DurgBank, 2023). Figure 2 depicts a pictorial representation of conventional and novel formulations.

Though most nanomedicines are the ones which are still in research or in the clinical trial phase and have not reached the market yet, the huge amount of data available proves that the extensive research in the field of nanomedicines will definitely bring about a revolutionary change in the treatment methodologies in near future.

### 3.1 Nano technology based-drug delivery systems in breast cancer therapy

Nanotechnology has brought about varieties of nanoformulation on the same platform, and which includes nanoparticles, liposomes, nanoemulsion, polymeric micelles dendrimers, etc. Nano formulations are the most emerging drug carriers with promising potential, i.e., controlled release effect of the drug (Byrne et al., 2008; Zhao et al., 2010; Xie et al., 2016), targeting of the pharmaceutical moiety to the desired site (Nobs et al., 2004)**,** bioavailability enhancement (Ahmad et al., 2019) of the therapeutic agent and enhancement of circulation time being the few notable examples. A suitably designed nanoformulation may possess any one or combination of these abilities, though it is also important to study the nature of the drug which will facilitate the synthesis/formation process. A perfectly designed novel drug delivery system can do wonders in treatment by altering the kinetics of the drug in the desired manner, and imparting plenty of advantages to the drug delivery when compared to those delivered through conventional dosage forms. Listed below is some information about the nanotechnology-based formulation which are either in the market or under research for the purpose of breast cancer management (Hussain et al., 2018; Du et al., 2019; Park et al., 2022; Cancer, 2023).

Nano emulsions are thermodynamically stable mixture of oil and water stabilized with appropriate mixture of surfactants and co-surfactants. These are classified as colloidal system in the submicron size range that serve as carriers for both hydrophilic and hydrophobic drug molecules. The droplet size varies from 10 to 1,000 nm (Hart et al., 2021)., mostly carrying a negative charge, except where the formulation is intentionally designed to be positively charged for specific reasons (Kerr et al., 2022). The droplet’s core consists of either water or oil, which gives it the property to act as a super-solvent for molecules that are hydrophobic and hydrophilic. These may be prepared by the high-pressure homogenization, microfuildization as well as ultrasonication (Kumar et al., 2019). The Lipid Nano Emulsions are prepared by the combination of oil and phospholipids with several benefits including high drug loading capacity, long-term stability, reduced irritability or toxicity of the incorporated drugs, reduced drug hydrolysis and no precipitation during administration. The submicron sized droplets of the Nanoemulsion promotes deposition and penetration of the active pharmaceutical ingredient to the target (Talegaonkar and Negi, 2015; Mahato, 2017; Burotto et al., 2019; Hart et al., 2021).

Nanoemulsion has been greatly exploited in drug delivery to breast cancer tumour (Nobs et al., 2004; Ganta et al., 2014). It exhibits better skin penetration due to low surface tension and the large surface area of the emulsion system (Ahmad et al., 2019). In addition, they may be considered as a substitute for liposomes and other vesicles as well (Park et al., 2022). Nanoemulsion are now-a-days considered as one of the promising formulations to achieve safe and effective cancer treatment (Verma et al., 2016; Nirmala et al., 2022). These formulations not only solve the problems related to water solubility issues (Verma et al., 2016; Barradas and de Holanda e Silva, 2021), but also can be customized for targeted cancer treatment (Song et al., 2020; Kaur et al., 2022).

The research in cancer therapy has become more focused on nanoemulsion due to its superficial charge (Migotto et al., 2018; Elena et al., 2019), enhanced half-life in blood circulation (Song et al., 2020; Kashyap et al., 2021), and large surface area (Yin et al., 2009; Gupta et al., 2016). Above all, nanoemulsion can easily accumulate on cancer tissue proving it to be one of the research turning points in cancer treatment through nanotherapeutics (Hussain et al., 2018).

Colloidal Dispersion at submicron range stabilized by surfactants is known as nanosuspensions. Nanosuspension can disperse hydrophilic drugs without any matrix suspended in dispersion enhancing the solubility of drugs. This approach of developing a nanosuspension as the formulation is useful for both poorly permeable and poorly soluble drugs. Above all, this formulation also renders dose reduction as well as enhancement of the physical and chemical stability of the drugs (Singh et al., 2017; Du et al., 2019; Pandey et al., 2020). Reports have proved that nanosuspension is also a well-researched formulation which can be convincingly used for the treatment of different malignant cells, i.e., Glioma and breast cancer respectively.

Liposome is a spherical bi-layered phospholipoidal nano-formulation having the property to encapsulate the fraction of solvent inside the core in which the solvent can easily float or diffuse.

Exclusively, liposome has the ability to carry both hydrophilic as well as lipophilic drug. The hydrophilic drugs are encapsulated in the internal aqueous core whereas; the lipophilic or hydrophobic drugs get embedded in the phospholipid bilayers. Simply by modifying the bi-layered composition of liposomes, the pharmacokinetics and *in-vivo* biocompatibility of the drugs can be improved. Yet another way of improving the liposomal formulation is by incorporation of PEG for enhancing the retention time of formulation in the systemic circulation. (Hofheinz et al., 2005; Kumar et al., 2019). This variant of the drug delivery system can enhance the duration of action by enhancing the circulation time of the drug for a prolonged period (Talegaonkar and Negi, 2015; Mahato, 2017). Pegylated liposomal Doxorubicin has been used as both combination chemotherapeutics and nanotherapeutics for efficient drug delivery in breast cancer management.

Liposomes are also effective in masking the unwanted toxic effects of various drugs as in the case of anthracyclines. Encapsulation in Liposome has promoted efficient drug cardiotoxicity and prolonged circulation time for effective drug delivery (Ganta et al., 2014).

The nanonization of drugs is getting considered due to a number of focal points which revolve around the conditions like dose reduction, improvisation of solubility, and enhancement of absorbance contrast with the crude form of the drug. The nanoparticles, after optimization usually have a size range of 10–100 nm (Ashfaq et al., 2017; Gavas et al., 2021). Those lying between this size range are considered to be most appropriate for cancer treatment (Smith et al., 2012; Jaiswal and Dudhe, 2015; Han et al., 2021). Nanoparticles can be classified into various types including metallic nanoparticles (Subhan, 2022), polymeric nanoparticles (Sartaj et al., 2021), solid lipid nanoparticles (Ashtari et al., 2020; Ozgenc et al., 2022), and fullerenes. The characterization of nanoparticles for drug release and drug targeting primarily depends on the evaluation of particle size and morphology (Song et al., 2020). Functionalized nanoparticles with ability of targeting complementary receptors are considered very effective in reducing side effects and enhancing efficacy of treatment (Gupta et al., 2016; Migotto et al., 2018; Elena et al., 2019; Kashyap et al., 2021; Kaur et al., 2022).

Nanogel is a modern nano-formulation where the nanoparticle is composed of hydrogel with an extremely crosslinked hydrophilic polymer. Nanogels are also defined as particles of the nanosize range formed by chemically or physically crosslinked networks of polymer that swells in a good solvent (Karthick et al., 2019). Nanogel systems have proved their efficacy in delivering drugs sustainably and in a targetable manner (Ahmad et al., 2019). Luo et al. (2020a) have developed gum Arabic aldehyde gelatin nanogels loaded with Curcumin for the treatment of breast cancer. The nanogel has been reported to improve the bioavailability and therapeutic efficacy of Curcumin in Breast cancer treatment (Luo et al., 2020a). There have been many other research studies that have proved that the use and acceptance of nanogel as a novel nanocarriers for the treatment of breast cancer among the scientific society of researchers (Park, 2002; Gavas et al., 2021). *In-situ* nanogel has opened a new avenue for long term sustained delivery of anticancer agents into the vicinity of breast tumor (Smith et al., 2012).

Small spherical micro particles, usually made up of biocompatible and biodegradable polymers having a size range of 1–1000 μm have the ability to encapsulate drugs in order to provide stability and enhance the therapeutic sustainability of the drug. Administration of medication through microencapsulation can be highly advantageous in various situations where the drug can be both ingested and injected depending on the need and desire (Subhan, 2022).

A quercetin-loaded PLGA microsphere are developed for the treatment of breast cancer. Similarly, there is a lot of research being conducted where microspheres have also been considered as one of the emerging possible carriers for drugs that can be used for the treatment of breast cancer (Sartaj et al., 2021).

Polymeric micelles have achieved noticeable results in the last few decades as a multifunctional nanotechnology-based delivery system for poorly water-soluble drugs. Hydrophobic part of the polymer forms the core of the polymeric micelle and the hydrophilic part of the polymer forms the corona. As a result the advantages of polymeric micelles as a delivery vehicle are two fold wherein, the hydrophobic core of the micelle assists in solubilization of poorly soluble drugs and the hydrophilic shell provides some protection in minimizing opsonin adsorption, longer blood circulation time to polymeric micelles and better blood stability. Owing to their smaller size polymeric micelles get concealed from scavenging by the mononuclear phagocytic system in the liver and circumvent the filtration of inter-endothelial cells in the spleen, contributing towards longer blood circulation time (Bhatia, 2016; Migotto et al., 2018; Ozgenc et al., 2022).

In addition, dendrimers, lipid nanoparticles (Ashtari et al., 2020), protein nanoparticles (Lin et al., 2009), ceramic nanoparticles, viral nanoparticles, metallic nanoparticles and carbon nanotubes are few more novel drug delivery systems which have been used in cancer therapeutics (Dhman, 2017). The Nanoparticles can be modified/functionalized in many ways to enhance drug localization, reduce opsonization, enhance circulation, enhance stability and bioavailability, reduce toxicity, increase drug efficacy and potentially decrease chances of multidrug resistance (Sarika and Nirmala, 2016; Liu et al., 2020).

### 3.2 US-FDA-approved drugs available in the market for the treatment of breast cancer to date

**FIGURE 3:**
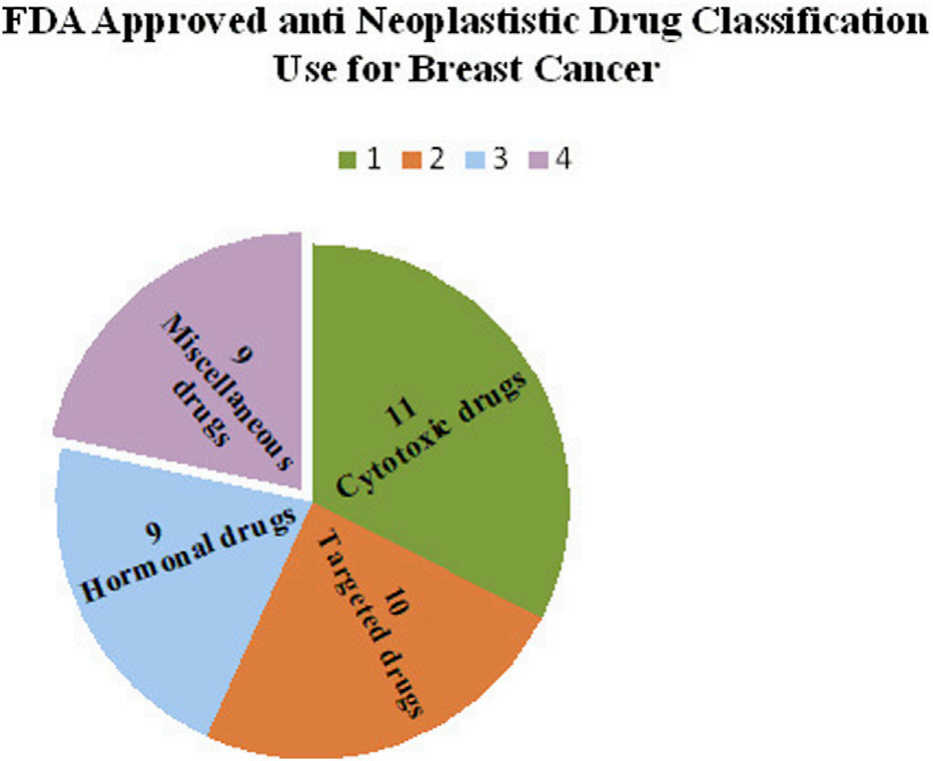
Relative Fractions of Drug classes approved by US-FDA for Different Breast cancer Indications.

**FIGURE 4:**
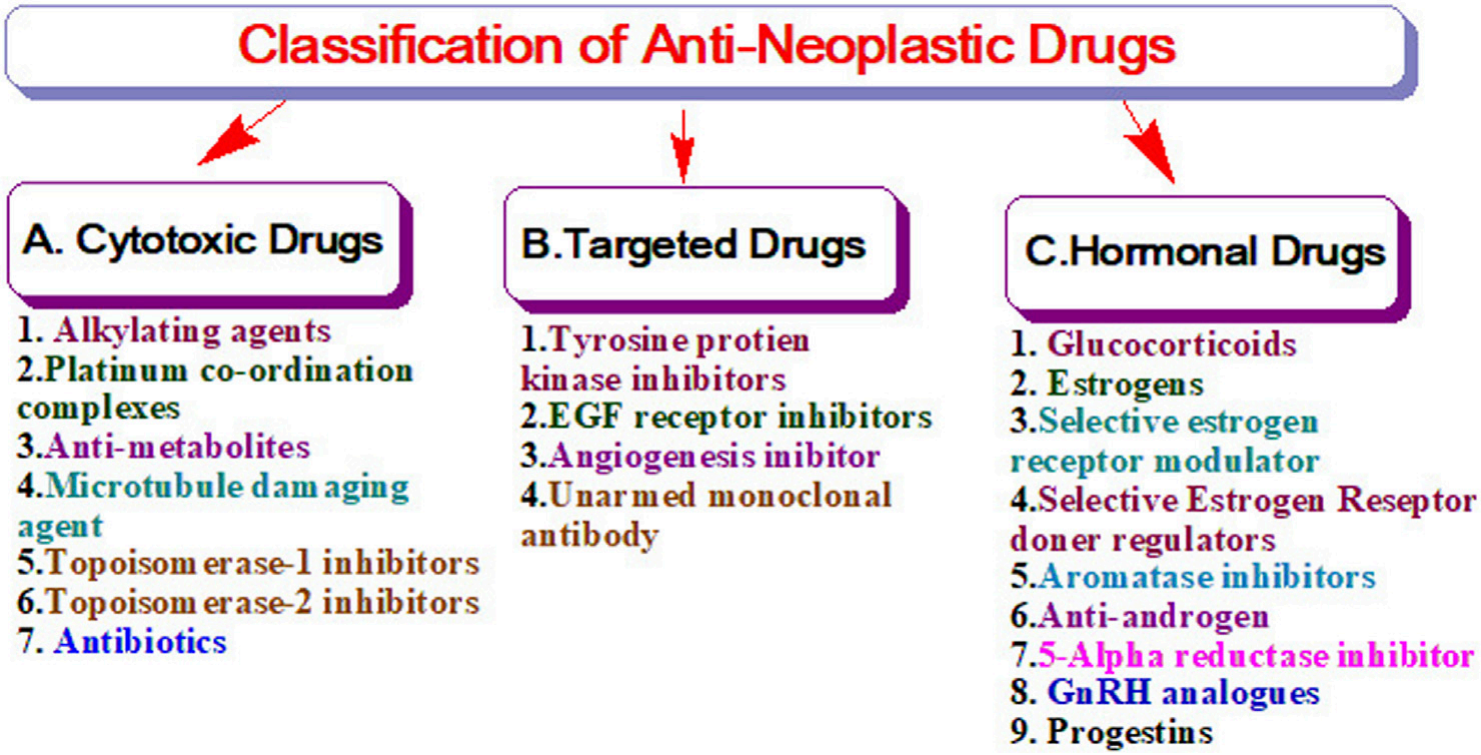
Classification of anti-neoplastic drugs.

The United States Food and Drug Administration (United States FDA) is a federal agency of the Department of Health and Human Services that regulates the approval process of drugs for various indications in humans and animals, along with various other products. Out of total 207 drugs which have been approved by FDA for Oncology, 39 are specifically approved for either a single or adjuvant treatment of breast cancer. In last 70 years FDA has approved more drugs for breast cancer indications than any other type of cancer (Sarika and Nirmala, 2016). The Figure 3 shows relative fraction of anticancer drugs from various categories which are approved for treatment of breast cancer. Major percentage of these drugs were approved for metastatic breast cancer at first. Later, approximately 31% of drugs received additional adjuvant status. According to a across sectional study published in year 2022 in JAMA Network Open Oncology, 19 drugs have been approved by USFDA between 31 May 2016 to 31 May 2021. However, most of these are for second, third and later line settings (Attama et al., 2022). This section summarizes all the listed and available drugs in the market being used for breast cancer. All the US-FDA-approved anti-neoplastic drugs have been discussed following the classification given in Figure 4. It contains information about the registered molecules, with emphasis on the date of their original approval, molecular targets, formulations and routes of administration and their indications in various forms of breast cancer.

#### 3.2.1 Cytotoxic drugs

The cytotoxic group of anti-neoplastic drugs consists of both cell cycle specific and non-specific agents which actively act against various cell cycle phases and hinders the growth of cells. These drugs mainly target the metabolic steps of cell division, so as to promote cell apoptosis. The cell cycle non-specific agents are not dependent on a particular phase of the cell cycle rather, it helps in hindering the cellular activity in all phases of mainly slow-growing tumors (Kircik, 2011). As depicted in Figure 4, these drugs are further sub-classified as nitrogen mustards, nitrosoureas and others. The upcoming sections summarize the details of all the drugs considered under the category of cytotoxic drugs used for breast cancer treatment.

##### 3.2.1.1 Cyclophosphamide

Cyclophosphamide is a nitrogen mustard alkylating agent which slows down the cancer cell growth by interfering with functioning of DNA. It was first approved in 1959 for use in malignant diseases including breast carcinoma (Cho and Moniri, 2016; Mirkes, 1985). Cyclophosphamide is a part of either combination or single treatment regime for breast cancer, administered either orally or through infusion, depending upon the need. It has the property of being inactive until metabolized by the liver. The active compounds Acrolein and Phosphoramide, slow cancer cell growth by binding and interfering with the actions of Deoxyribonucleic acid (DNA) within cancerous cells (Singh et al., 2017). Cyclophosphamide is not only available in the form of injection (like intramuscular injection, intra peritoneal injection, intra pleural injection) but also in the form of tablets where the active ingredient is present at a quantity of 25 mg and 50 mg for oral administration (Lin et al., 2009). Cyclophosphamide with methotrexate and fluorouracil has been considered an adjuvant chemotherapeutic regime for breast cancer management (Press, 2012). Being teratogenic in nature it is contraindicated in pregnant women (Yao et al., 2020; Benjamin et al., 2022). Adverse reactions viz. Bone marrow suppression, alopecia, nausea, and hemorrhagic cystitis to cyclophosphamide are due to its cumulative administration. It should be cautiously administered to patients with heart, kidney, and pulmonary diseases. Cyclophosphamide (Cyp) is used in high doses and in combination with other drugs (Pharmacology, 2021). In an attempt to reduce the off-target effects and enhance the therapeutic efficacy (Rao et al., 2016; Tiash and Chowdhury, 2016), Snigdha Tiash and Md Ezharul et al. (2016) prepared pH-sensitive carbonate apatite nanoparticles. The anticancer capacity of Cyclophosphamide is enhanced upon its encapsulation within citric acid dendrimers. Citric acid dendrimers impart solubility and specificity to the drug (Burn, 1961).

##### 3.2.1.2 Tepadina

Thiotepa also known as Tepadina is an organophosphorus alkylating antineoplastic agent approved in 1959 was used to treat a variety of solid and hematologic malignancies. This drug also carries a US Food and Drug Administration (FDA) indication for the treatment of breast adenocarcinoma in the recommended dose of 0.3–0.4 mg/kg intravenously at 1–4 weeks intervals (FDA, 2012c).

This chemical entity causes interference in DNA replication as well as cell division by the formation of cross-linkages between alkylated guanine bases resulting in apoptosis and cell growth inhibition in tumor cells (Zhang et al., 2003). The most common adverse reactions with greater than 10% incidences are neutropenia, anemia, thrombocytopenia, elevated alanine aminotransferase, elevated aspartate aminotransferase, elevated bilirubin, mucositis, cytomegalovirus infection, hemorrhage, diarrhea, hematuria, and rash (Comte et al., 2013). It should be used cautiously with pregnant women and patients with hepatic and renal impairment. Its commercially available as a single dose, lyophilized white powder for reconstitution (FDA, 2012c; FDA, 1959). Novel formulation, i.e., gelatin microparticles with a diameter of approximately 2 μm, and loaded with Thiotepa were prepared through a chilled dehydration procedure. The size of formulation at least doubles the AUC, suggesting that the dose might be halved, thereby reducing side effects associated with this otherwise important drug. Recently, an Iron-Doped Fullerene Cage of Thiotepa has been prepared by Xuan Young to modify drug delivery (Alemrayat et al., 2018; Pandey et al., 2020). C3 N nanotubes (Ge et al., 2012; Torabifard and Fattahi, 2012; Fox et al., 2021). Loaded with Thiotepa has been tried in combination with ifosfamide, etoposide, and rituximab (TIER) for the treatment of PCNSL relapsed or refractory to high-dose methotrexate-based chemotherapy (Kumar et al., 2019).

##### 3.2.1.3 Methotrexate Sodium

Methotrexate is highly exploited antimetabolite antineoplastic agent which acts as a stoichiometric inhibitor of dihydrofolate reductase. It is a structural analog for folic acid due to which it can be used to target the over-expressed folate receptors on the tumor cells. It was approved for breast cancer indication by FDA in 1959. Apart from breast cancer, Methotrexate Sodium is also effective against head and neck cancer, leukemias, lymphomas and carcinomas (Syn et al., 2017). Reditrex, an injection for subcutaneous administration was the first approved formulation which is marketed as prefilled syringes in strengths ranging from 7.5 mg to 25 mg. Patients on MTX therapy should be closely monitored for liver, lung, skin, and kidney toxicities and bone marrow suppression (Mirkes, 1985; Li et al., 2012). Various MTX-loaded nanocarriers have been reported with significance in the treatment of different cancers types including breast cancer. Lipid nanoemulsion (Talegaonkar and Negi, 2015), Nanogel (Mahato, 2017), and (Ganta et al., 2014), hydrogel (Han et al., 2021). Solid lipid nanoparticles (Vuong et al., 2022). Nanosuspension (Accessdata, 2019; Li et al., 2022), niosomes (Moura et al., 2011; Avasatthi et al., 2016), liposomes (Kumar et al., 2004; Agrawal et al., 2020), etc. are few extensively exploited nano-systems. MTX in the nano-formulations indicated high mean residence time in blood circulation that helps them to accumulate at the desired targeted sites.

##### 3.2.1.4 5-Flourouracil

5-Fluoro-1, 3-Diazinane-2, 4-Dione or 5-Flourouracil is the most potent and successful first-line medication treatment for cancer. It is thought to be formed by the covalent binding of the drug, deoxyribonucleotide (FdUMP) and the folate cofactor, N5-10- methylenetetrahydrofolate, to thymidylate synthase (TS) (Battaglia et al., 2017). This inhibits the formation of Thymidylate from Uracil, resulting in the inhibition of DNA and RNA synthesis and causing Thymine less cell death. Fluorouracil can also be incorporated into RNA in place of Uridine Triphosphate (UTP), resulting in a bogus RNA and interfering with RNA processing and protein synthesis (Li et al., 2019). 5-Fluorouracil is widely used for colorectal cancer and was later approved for breast cancer treatment as well. It has been reported to cause fogging and memory impairement, when used for long term in breast cancer (Powar et al., 2021). Similar to Methotrexate nearly all types of novel formulations have been researched for 5-FU. Few recently reported nano-systems are; 5-Fluorouracil formulation in Nanoporous Biogenic Mg-calcite from Blue Crab Shells (Trotta et al., 2004; Horo et al., 2019; Lazar et al., 2021), a photo-responsive chitosan conjugated pro-drug nano-carrier for controlled delivery (Horo et al., 2019), Folate-tagged chitosan-functionalized gold nanoparticles (Longley et al., 2003; Akinyelu and Singh, 2019), 5- fluorouracil-loaded calcium phosphate nanoparticles (Bhadra et al., 2003), Folic acid-navigated and β-cyclodextrin-decorated carbon-encapsulated iron nanoparticles (Wigmore et al., 2010; Kasprzak et al., 2018), folate receptor targeted nanoliposomes (Handali et al., 2019), controlled release PVC/PEG polymeric films, amine functionalised hollow mesoporous silica nanoparticles (HMSN-NH2) and then coated with a biocompatible polydopamine (PDA) (Cheralayikkal et al., 2022), multiple-nano-emulsion etc (Bhadra et al., 2003).

##### 3.2.1.5 Capecitabine

Capecitabine is an oral fluoropyrimidine which is preferentially converted to fluorouracil in tumor tissues in a three-step enzymatic cascade. The final stage of conversion to fluorouracil is catalyzed by thymidine phosphorylase, which is appreciably more active in tumor than in healthy tissues (Radwan et al., 2012), It is a common treatment medicine for HER-2 positive Breast cancer. It is presently the only active treatment regime specifically approved for those cancer patients where the tumor is absolutely resistant to any other treatment regime including Paclitaxel and Anthracyclines. It has been reported that on exposure to Capecitabine cancer cells undergoes apoptosis and cell death (Summary, 2005; Cheralayikkal et al., 2022).

##### 3.2.1.6 Vinorelbine Bitartrate

Vinorelbine Bitartrate is a semi-synthetic derivative of Vinca alkaloid present in the dried leaves of vinca rosea. It is mainly characterized as poisons for spindle formation, hence, known as mitotic spindle poison. It has been reported to interfere in the polymerization of tubulin protein which is involved in the process of cell division. (Ahmad et al., 2022). Vinorelbine has been widely accepted to treat solid tumors, such as non-small cell lung cancer. It was approved for breast cancer indication in 1990 (Radwan et al., 2012). There have been studies which has tested the efficacy of Vinorelbine alone, or in combination with other drugs so as to develop a therapy that is non-cross resistant with Taxanes and Anthracyclines. Vinorelbine alone or in combination is an effective and safe treatment for pre-treated locally advanced, metastatic, or secondary breast cancer patients. When combined with BET inhibitors, metastatic breast cancer in the brain become more sensitive to Vinorelbine (Summary, 2005). with cyclophosphamide, Vinorelbine activate Stem-Like CD8 + T Cells and Improve Anti-PD-1 Efficacy in Triple-Negative Breast Cancer (Walko and Lindley, 2005; Petrelli et al., 2016). It is available in the market as injectable and oral formulations. Few novel formulations, i.e., liposome (Marcucci and María, 2004), transdermal based hydrogel formulations (Fonseca et al., 2021), lipid-based nano-formulation (Cybulska-Stopa et al., 2013; Kanojia et al., 2020). Have also been researched. However, injection is still the only commercially available form.

##### 3.2.1.7 Docetaxel

Docetaxel is the first line of chemotherapeutic among Taxane group of drugs which was approved by the US-FDA in 1996 for the treatment of breast cancer (Falvo et al., 2021). Used at the dosage of 60 mg/m^2^ to 100 mg/m^2^ as a single agent and in 75 mg/m^2^ in combination with Doxorubicin and Cyclophosphamide. Adjustment of dosage is decided by the appearance of the side effects, i.e., febrile neutropenia, neutrophils and lt; 500 cells/mm 3 for more than 1 week, or severe or cumulative cutaneous reactions. It is strictly prohibited for the patient with impaired liver functions and reported hypersensitivity with DTX or polysorbate formulations. Patients who are recommended DTX therapy are pretreated with oral corticosteroid (e.g., Dexamethasone, 16 mg/day) for 3 days to reduce fluid retentions (Falvo et al., 2021). Docetaxel binds to the tubulin-subunit. Tubulin is a building block of microtubules, and Docetaxel binding secures these building blocks. The resulting microtubule/Docetaxel complex is unable to disassemble. This has a negative impact on cell function because the dynamic instability of microtubules is required for their function as a transportation highway for the cell (Marcucci and María, 2004; Ahmad et al., 2014). The biggest disadvantage of Docetaxel is that it leads to chemotherapeutic resistance which can even result in the relapsing of tumors. Challenges with administration during mixing of the docetaxel with the diluent and need of targeting necessitated remodeling of the currently available docetaxel formulation but none has made it to clinical setting as an alternative. A recently published review by Karamot and Oyediran summarized virtually all the novel formulations researched on Docetexal (Fonseca et al., 2021).

##### 3.2.1.8 Doxorubicin Hydrochloride

Doxorubicin Hydrochloride is the cytotoxic antibiotic derivative of Anthracycline obtained from *Streptomyces* Peucetius micro-organism. (Drug bank, 2005; Bahadori et al., 2014). Its primary mechanism is to intercalate within DNA base pairs by inhibiting the Topoisomerase II enzyme, causing breakage of DNA strands and inhibition of both DNA and RNA synthesis. Cell treated with DOX manifests many morphological changes which leads to apoptosis and is thought to be responsible for therapeutic use. When combined with iron, Doxorubicin also causes free radical-mediated oxidative damage to DNA, further limiting DNA synthesis. Currently, Doxorubicin is used for the treatment of metastatic and orthotropic breast cancer (Luo et al., 2020b). In practice, it is administered in a single dose of 60–75 mg/m^2^ in intervals of 21 days as i.v. injection which may be reduced to 40–60 mg/m^2^ when given as a combination. Administration should be slow and in the large vein with saline running in the tubing. Injection should be immediately stopped if any extravasation is suspected (Hanna et al., 2014; Bland et al., 2019). It should not be co-administered with any cardiotoxic drug, and in case of concomitant administration, elimination of previously administered dug is to be ensured. Lipodox, Evaset, Doxil/Caelyx, Myocet are Liposomal formulations of Doxorubicin Hydrochloride. These formulations had market of 1 billion USD in 2020 and is forecasted to have increased to 1.39 billion USD by 2025. The novel formulations are still being tried for DOX. Martina Di Francesco prepared Doxorubicin Hydrochloride-Loaded Nonionic Surfactant Vesicles to Treat Metastatic and Non-Metastatic Breast Cancer (Di Francesco et al., 2021).

##### 3.2.1.9 Paclitaxel

Paclitaxel is a natural product with antitumor activity. TAXOL (paclitaxel) is obtained via a semi-synthetic process from Taxus Baccata. Paclitaxel is a white to off-white crystalline powder that is highly lipophilic, insoluble in water, and melts at around 216–217°C (Oyediran et al., 2022).it is widely used clinically for the treatment of various types of tumors such as breast (Patel, 1996)., pancreatic, cervical, ovarian, etc. Paclitaxel has been utilized in combination therapy with other drugs, or it is also considered as a first line. Paclitaxel emerged as an important agent for breast cancer treatment because of its lack to Anthracycline cross resistance and tolerability towards clinical investigation, though later Paclitaxel was reported with several adverse effects like cardiovascular toxicity, undesirable gastrointestinal effects, bone marrow suppression, etc (Di Francesco et al., 2021). It is marketed as multidose vial packaged in an individual carton. Various strengths available are NDC 0015-3475-30 30 mg/5 mL, NDC 0015-3476-30 100 mg/16.7 mL, and NDC 0015-3479-11 300 mg/50 mL diluted to a final concentration of 0.3–1.2 mg/mL, prior to administration. The treatment may cause Anaphylaxis and severe hypersensitivity reactions. Hence, it should be strictly monitored by experts. Nab-Paclitaxel Nab-Paclitaxel is basically nanoparticle albumin-bound paclitaxel which is a novel marketed nano-formulation showing better anti-neoplastic activity with less toxic effect than the normal solvent-based paclitaxel (Hanna et al., 2014; Alves et al., 2017). This drug is marketed as a 100 mg injection in the name of Abraxane. Lipusu™ and Genexol are other marketed forms of PTX in non-conventional forms (Du et al., 2018). Various reviews published from time to time have listed various novel formulations which have been prepared for Paclitexal to overcome the limitations with present therapy (Marupudi et al., 2007; Yamamoto et al., 2011; Chatterjee et al., 2017).

##### 3.2.1.10 Eribulin Mesylate

Eribulin Mesylate was approved in 2010 by US FDA for metastatic breast cancer (Singla et al., 2002). A patient who received at least 2 chemotherapeutic regimens for metastatic cancer (Of and Information, 2010). (Prior therapy should have included an anthracycline and a Taxane) (Mcbride and Butler, 2012) was treated with an injection of Eribulin Mesylate that is administered as 1.4 mg/min intravenously over 2–5 min on Days 1 and 8 of a 21-day cycle. FDA Reference ID: 2863825. Eribulin inhibits microtubule growth without affecting shortening and sequesters tubulin into nonproductive aggregates. Eribulin acts through a tubulin-based antimitotic mechanism, causing G2/M cell-cycle blockage, mitotic spindle disruption and, eventually, apoptotic cell death after prolonged mitotic blockage (Jain and Vahdat, 2011). Importantly, Eribulin also had an acceptable toxicity profile and therapeutic window in mice across several dosing schedules (Dybdal-Hargreaves et al., 2015).

##### 3.2.1.11 Epirubicin

Epirubicin is the epi-isomer of the anthracycline antibiotic named Doxorubicin (Markes et al., 2006). It is basically a semi-synthetic derivative of Doxorubicin which was first approved in France in 1982. It is used extensively for the management of breast cancer as adjuvant therapy for early breast cancer patients. (Levine, 2000). Epirubicin intercalates into DNA by topoisomerase II inhibition. This ultimately leads to oxygen generation and interferes in the protein synthesis of tumour cells hence the cell growth is stopped. This agent also produces toxic free-radical intermediates and interacts with cell membrane lipids causing lipid peroxidation (Ormrod et al., 1999). It is recommended in doses of 100–120 mg/m^2^ administered as an intravenous bolus. Administration is recommended in two types of regimens with 5-Fluorouracil and Cyclophosphamide. Therapeutic doses of Epirubicin may cause tissue necrosis, cardiac toxicity, secondary acute myelogenous leukemia, and myelosuppression (Conte et al., 2000). Description. However, in clinical trials, lesser non-hematologic and cardiac toxicities are reported for equimolar doses of Epirubicin than Doxorubicin (Epirubicin, 1992; FDA, 1999). Biocompatible Polymer PLA–PEG–PLA Nanoparticles (Massadeh et al., 2021; Sheydaei, 2021), polymeric nanoparticles (Torchilin, 2007), Polymeric miscelles (FDA, 1998), long-circulating thermo sensitive liposomes are few prominent newer efforts made for better delivery profile of Epirubicin.

#### 3.2.2 Targeted drugs

**FIGURE 5:**
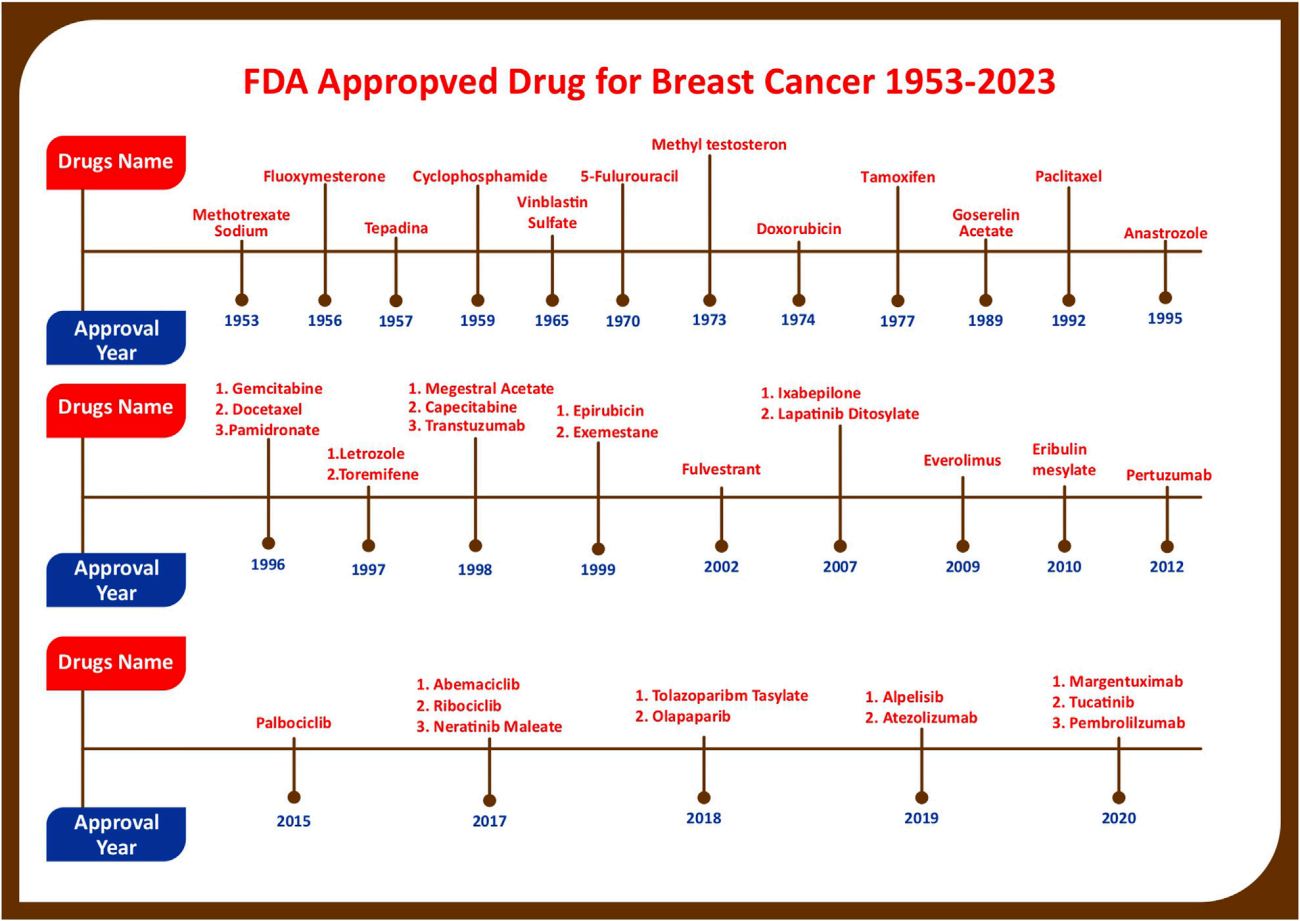
Figure showing progress in breast cancer treatment research as year wise account of anticancer drugs which are approved for various Breast Cancer Indications by US-FDA.

Targeted drugs mainly work by selectively interacting with the protein receptors that control the growth, differentiation, and migration of cancerous cells or reactivating Programmed cell death which is otherwise compromised in some types of Breast Cancers. Targeted anti-neoplastic drugs are considered to be the foundation for the future of precise oncology. As they are thought to be devoid of unintended side effects on healthy cells, which otherwise is the case with virtually all other classes of anticancer drugs. Most of the targeted drug molecules are either small molecule drugs or monoclonal antibodies. The biggest disadvantage of targeted therapies is the development of drug resistance. The resistance can happen due to the morphological or physiological change in the receptors after being exposed to the same chemical moiety for a longer period of time. As depicted in Figure 5, these drugs are sub-classified as monoclonal antibodies, tyrosine kinase inhibitors as mentioned in other classes.

##### 3.2.2.1 Lapatinib ditasylate

Lapatinib is a selective and potent reversible Tyrosine Kinase Inhibitor that is considered to inhibit human epidermal growth factor receptors (HER-2) overexpressed in breast cancer cells (Soiza et al., 2018). It targets the intracellular ATP- binding sites of the HER-2 receptors, binds competitively, and hinders cell growth. Lapatinib is administered as a tablet in combination with Capecitabine in patients who have received prior therapy including an Anthracycline (Shantanam, 2018). It can be moderately well tolerated when administered orally once every day in daily doses of 1,250 mg–1,500 mg for 1–21 days. Gastrointestinal complications and skin rashes are the main toxicities observed in more than 20% of patients consuming Lapatinib (Yuan and Xu, 2021). It has been reported that Lapatinib is well accepted in patients who are resistant to Trastuzumab. It has shown improvement in the overall survival period of breast cancer patients (Alemrayat et al., 2019). Clinical use is limited due to its poor aqueous solubility, poor bioavailability, high binding affinity toward blood proteins, and toxicities related to its higher dose, and stability. Various nano-delivery systems, including nanoparticles, polymeric micelle, core-shell nanoparticles, nanochannel, were investigated to overcome these issues (Emens et al., 2021).

##### 3.2.2.2 Transtuzumab

Transtuzumab was the first Monoclonal Antibody approved by US-FDA in 1998 for the treatment of HER2 overexpressing breast cancer and the treatment of HER2-overexpressing metastatic gastric or gastroesophageal junction adenocarcinomas (Guerreiro et al., 2016; Yassemi et al., 2020). Transtuzumab selectively binds with overexpressed HER-2 receptors, inducing an immune-mediated response that causes internalization and recycling of HER-2. It may also upregulate cell cycle inhibitors such as p^21^ and p^27^ (Murthy et al., 2020; Khan et al., 2022). It is marketed under the brand name HERCEPTIN^®^ as IV infusion and is administered in two types of regimens, weekly and three weekly regimens and is also on the list of essential drugs by WHO. Though there are several concerns related to cardiotoxicity and the development of resistance that remain in consideration, besides several other therapeutic issues also remain unclear and have been addressed in an inconsistent way. The main reason behind this is that there is still a lot of information to be documented in the scientific literature on pharmaco-dynamics, pharmaco-kinetics, and clinical use of the drug (Kelly and Buzdar, 2010). Transtuzumab is considered to be the golden standard of treatment for this sub-type of breast cancer. Research work is being done on use of trastuzumab in novel formulations (Barradas and de Holanda e Silva, 2021).

##### 3.2.2.3 Margetuximab

US-FDA approved Margetuximab in the year 2020 as a combination chemotherapeutic for use in adult patients suffering from metastatic HER-2 positive breast cancer. This is recommended for only those patients who have already received two anti-HER 2 treatment regimens prior to this (Goss and Tye, 1997). The Margetuximab is a chimeric antibody that binds to the extracellular domain of the human epidermal growth factor receptor 2 protein (HER2) (Markham, 2021; Rugo et al., 2021), inhibits tumor cell proliferation, reduces shedding of the HER2 extracellular domain, and mediates antibody-dependent cellular cytotoxicity (ADCC). Margetuximab shares ERBB2 specificity with Trastuzumab but incorporates an engineered Fc region for increased binding to activating Fcγ receptor IIIA (CD16A) and decreased binding to inhibitory Fcγ receptor IIB (CD32B) relative to Trastuzumab with the aim of improving response rates (Barros-Oliveira et al., 2017). From Phase 3 Randomized Clinical Trial Hope S et al. concluded that Margetuximab plus chemotherapy had an acceptable safety and a statistically significant improvement in PFS compared with Trastuzumab plus single agent chemotherapy in ERBB2-positive ABC after progression on 2 or more prior anti-ERBB2 therapies (Sanford and Plosker, 2008). Unlike other monoclonal antibodies used for treatment of breast cancer, Margetuximab is administered through IV route and is available as 250 mg/10 mL single dose vial.

##### 3.2.2.4 Atezolizumab

Atezolizumab is a humanized, Fc optimized, monoclonal antibody. It has received accelerated approval in March 2019 for Triple-Negative Breast Cancer that has spread or cannot be removed by surgery and tests positive for “PD-L1” (Alyafee et al., 2018; Aleem and Shah, 2023). Atezolizumab kills the cancer cells by blocking the interaction of PD-L1 receptors with PD-1. Hence, preventing the blocking of inhibitory signals to killer cell activation and reactivating programmed cell death (Structures et al., 2023). It is used in combination with the Abraxane (not with Paclitexal). It is manufactured and marketed by Genentech in United States under the name of Tecentriq. It has, however, been reported that in October 2021 the company has withdrawn Tecentriq voluntarily from patients who were under its treatment regimen in the United States, though the withdrawal does not affect the approval for other countries where this drug is used for the treatment of metastatic PD-L1 positive of triple-negative breast cancer (Untch and Jackisch, 2008). The patient may suffer Immune-Mediated Adverse Reactions along with common adverse reactions which may prove fatal (Jayapal and Dhanaraj, 2017).

#### 3.2.3 Tyrosine-protein kinase inhibitors

Until recently, the mainstay of treatment in the majority of hormone receptor (HR)-positive, human epidermal growth factor 2 receptor (HER2)-negative advanced breast cancer (ABC) consisted of single-agent endocrine therapy (ET). However, as the understanding of endocrine resistance has grown, newer targeted agents have come to the fore (Drugbank, 2023).

##### 3.2.3.1 Abemaciclib

Abemaciclib is an anticancer moiety marketed as Verzenio among many others. It has received initial approval in 2017 by US-FDA for the adjuvant treatment of adult patients with HR-positive, HER-negative, and node-positive early Breast Cancer in combination with endocrine therapy (Tamoxifen or an aromatase inhibitor). With aromatase inhibitors and Fulvestrant Abemaciclib is recommended for HR-positive, HER-negative metastatic (McCartney et al., 2018), and advanced breast cancer. Abemaciclib inhibits CDK4 and CDK6 causing inhibition of phosphorylation of the retinoblastoma protein (Rb), and cell cycle progression from G1 to S, and cell proliferation. Ultimately cell death by apoptosis. Currently, it is available as an oral tablet formulation in two strengths 150 mg, and 200 mg.

##### 3.2.3.2 Alpelisib

Phosphoinositol-3- Kinase (PIK-3) is a group of enzymes that are involved in cell growth, cell differentiation, and proliferation. Activation of the Phosphatidylinositol-3-Kinase (PI3K) pathway via PIK3CA mutations occurs in 28%–46% of hormone receptor-positive (HR+), human epidermal growth factor receptor-2-negative (HER2-) advanced breast cancers (ABCs) and is associated with poor prognosis (Cheer et al., 2005; Sullivan et al., 2022). Its inhibition becomes significantly important for the treatment of cancers like advanced or metastatic breast cancer. Alpelisib is an oral Kinase inhibitor that selectively inhibits mutated phosphoinositol-3 kinase (PIK-3). It has been approved by US FDA in 2019. Its indications include post-menopausal women, men, HR-positive and HER-2 negative, and PIK3CA mutated metastatic breast cancer (Cheer et al., 2005; André et al., 2021). The drug is taken orally and administered in combination with Fulvestrant (Papich, 2021). It is marketed under the brand name Piqray among many others. Available in 3 different dose sizes and administered OD. Diarrhea is a common adverse event associated with the use of Alpelisib. However, during post-marketing surveillance, Kathleen and co-workers observed colitis as a new safety signal (Gregory et al., 1985). It is also approved for medical use in Australia and European Union also.

##### 3.2.3.3 Palbociclib

Palbociclib is a reversible inhibitor of Cyclin-Dependent Kinase-4 (CDK-4) and Cyclin-Dependent Kinase-6 (CDK-6) like Abemaciclib and Alpaciclib. Palbociclib received approval from US-FDA in 2015 for the treatment of women who are in their post-menopausal phase suffering from HER-2 negative and ER-positive breast cancer (Sledge et al., 2020). It has shown to improve overall survival in Hormone Receptor–Positive, ERBB2-Negative breast cancer significantly which otherwise progressed on Endocrine Therapy (Bayraktar et al., 2013). In combination with an aromatase inhibitor similar to the other two kinase inhibitors. (Bowles et al., 2015; Houdaihed et al., 2018; Neven et al., 2021). The drug is administered orally and is available in the market in form of capsules. The recommended dose is 125 mg daily for 21 days. However, capsules are available in three different strengths; 75 mg 125 mg as dosage adjustment may be recommended in special cases of dose dependant side effects, kidney and liver disease, and pregnancy state of female patients.

##### 3.2.3.4 Neratinib Maleate

Neratinib is a 4-Anilino-3-Cyano Quinoline derivative, formulated as tablets as Neratinib Maleate (FDA, 2017a). Neratinib is a pan-HER, irreversible TKI with potent preclinical activity against Trastuzumab-resistant breast cancer models (Yin et al., 2016). Neratinib binds irreversibly to different epidermal growth factor receptors like HER2, HER4 and EGFR receptors, reduces the autophosphorylation of the receptors which creates a barrier for the down-streaming of the signal pathway. This exhibits antitumor activity. Neratinib received US-FDA approval in 2017 for the patient with early-stage breast cancer (as single agent adjuvant) and advanced or metastatic HER2-positive breast cancer (in combination with Capacitabin) (Saura et al., 2020). It is available in market as 40 mg film coated tablet for oral use. Most common side effects observed with Neratinib are Diarrhoea, followed by nausea, vomiting, abdominal pain and anorexia. Based on several studies, it is likely that Neratinib-related diarrhea is caused by HER1/EGFR inhibition (Yin et al., 2016).

##### 3.2.3.5 Tucatinib

Tucatinib turned out to be the first chemical moiety which was evaluated under Project Orbis which is an FDA oncology Center of Excellence initiative. Tucatinib was approved by FDA in 2020 for a combination therapy with Trastuzumab and Capecitabine for the treatment of unresectable advanced or metastatic breast cancer with brain metastases (Silvestris et al., 2008). Tucatinib is a Tyrosine Kinase inhibitor of HER-2. Hence, it inhibits the growth of HER2 expressing tumors. The combination of Tucatinib and Trastuzumab showed increased anti-tumor activity *in vitro* and *in vivo* compared to either drug alone and had acceptable toxicity (Elith* et al., 2006). It is marketed as tablets, for oral use in two strengths.

##### 3.2.3.6 Pertuzumab

Pertuzumab, a monoclonal antibody against HER2, was approved by the FDA in 2012 for the treatment of patients with HER2-positive MBC who had not previously received anti-HER2 therapy or chemotherapy for metastatic disease. The approval was based on the CLEOPATRA trial, which included patients with HER2-positive MBC who received Trastuzumab and Docetaxel in combination with either placebo or Pertuzumab. The addition of Pertuzumab resulted in a significant improvement in the primary endpoint of PFS. The final OS analysis also revealed a statistically significant benefit for the Pertuzumab arm. This was the first approval after trastuzumab in more than a decade for an antibody targeting HER2 that showed a survival benefit when combined with Transtuzumab.

#### 3.2.4 Hormonal drugs

The breast cancer cells get attracted to hormones like estrogen, progesterone through the specific receptors present on the cells which help them to grow. The treatments to stop these hormones from interacting with the receptors are known as hormone therapy. Hormone therapy mainly revolves around the concept of steroidal hormones and their receptors on the breast cancer with reference to the mechanism of ligand-receptor interaction (Abdulkareem and Zurmi, 2012; US Food and Drug Administration, 2017).

##### 3.2.4.1 Tamoxifen

Tamoxifen (TAM) is a hydrophobic estrogen modulating anticancer agent, approved by the US-FDA for treatment of breast cancer through hormone therapy. Tamoxifen acts as a selective estrogen receptor modulator (SERM) for estrogen receptors. It acts as an anti-estrogen agent for breast cancer cells whereas, performs the function of estrogen agent for normal cells and tissues. Tamoxifen is a specific antagonist for ERα and thus shows an antiproliferative effect (Hortobagyi, 2018; Hong et al., 2020).

##### 3.2.4.2 Toremifene

Toremifene works as a selective non-steroidal triptycene estrogen receptor modifier that is being used for a long time in hormone receptor positive breast cancer of both late and early stage. Toremifene is metabolized in the liver and is excreted out from the body through feces. It is also commonly used in clinical practice as an alternative to Tamoxifen. Both tamoxifen and teromifene are structurally similar drugs which are commonly used for endocrine therapy after breast cancer surgery (Mustonen et al., 2014; FDA, 2017c). It is also teste for neoadjuvant therapy for locally advanced breast cancer in combination with Melatonin or Metformin (Sartaj, AnnuBiswas, Verma, Sahoo, Baboota, et al.). A research work published in International Journal of Cancer (2018) claims that Toremifene, rather than Tamoxifen, might be a better option for the adjuvant endocrine therapy in CYP2D6*10T/T genotype breast cancer patients in (Ahmed et al., 2022; Mazzarino et al., 2013). Another prospective, randomized study of Toremifene vs tamoxifen for the treatment of premenopausal breast cancer shows TOR and TAM have similar side effects on the female genital system and quality of life in premenopausal early breast cancer patients (Fei and Yoosefian, 2021).

##### 3.2.4.3 Fulvestrant

Fulvestrant is a selective degrader of estrogen receptor that first binds with the estrogen receptor and acts as an inhibitor for estrogen signaling which fuel the process of tumor cell growth. Unlike Tamoxifen, Fulvestrant-induced conformational change of estrogen receptors hinders transcriptional activity of proteins. In addition, the unstable complex formed during the interaction of Fulvestrant with estrogen receptor results in accelerated degradation of the cells. Fulvestrant therefore acts as both a competitive antagonist and a selective estrogen receptor degrader (SERD), causing a reduction in cellular estrogen receptor alpha levels. Fulvestrant is drug which is recommended as a monotherapy but there are still multiple trails going on observing its efficacy in combination therapy as a CDK4/6 inhibitor (Bowles et al., 2015; Soiza et al., 2018), (Kabos and Borges, 2010; Shantanam, 2018). Despite its increasing use in the ER + metastatic breast cancer setting, data are available in the literature about its-acquired endocrine resistance (Robson et al., 2017).

##### 3.2.4.4 Letrozole

Diminishing estrogen production by antagonizing the conversion to estrogen from androgens is achieved by aromatase inhibitors. The main function of aromatase inhibitors is to antagonize the activity of aromatase enzyme, in turn resulting in inhibition of estrogen production in breast cancer patients. Letrozole was developed as a highly potent third generation non-steroidal inhibitor of aromatase used for the treatment of breast cancer management. In several preclinical studies, Letrozole has demonstrated greater potency compared with Anastrozole, Exemestane, Formestane and aminoglutethimide. Letrozole has been reported to inhibit the aromatase activity by more than 99% in *in-vivo* tissues. It has been documented to exhibit its most wide application in recurrent, metastatic and advanced cancer in post-menopausal women. Unlike first and second-generation Aromatase Inhibitors, Letrozole is highly selective for aromatase and does not significantly affect 17a-OH progesterone, cortisol aldosterone, thyroxine, thyroid-stimulating hormone (TSH), luteinizing hormone (LH), follicle-stimulating hormone (FSH) or androstenedione (Holmberg et al., 1997; Clinicaltrials.Gov, 2015; Rugo et al., 2019). It has poor water solubility, rapid metabolism, and a range of side effects. Polymer-based nanoparticles (FDA, 2012a; Alemrayat et al., 2018; Caulfield et al., 2019; Leo et al., 2020), Lipid nanocomplex (Bhatnagar, 2007; Azandaryani et al., 2019; Yassemi et al., 2020), solid lipid nanoparticles (Khan et al., 2022), Pegylated nanoparticles (Kelly and Buzdar, 2010), are few of the novel formulations explored to overcome the limitations and side effects associated with Letrozole.s.

##### 3.2.4.5 Anastrozole

Anastrozole was first approved in United States, EU and in other countries as aromatase inhibitor for adjuvant treatment in postmenopausal women suffering from hormone-positive early-stage breast cancer (Goss and Tye, 1997; Ekedahl et al., 2022). It is generally well-tolerated in patients suffering from early-stage breast cancer. Aromatase inhibitors evolved as an alternative of endocrine therapy for the treatment of hormone sensitive breast cancer. Anastrozole is a potent non-steroidal aromatase inhibitor which selectively blocks estrogen synthesis in women having breast cancer who are in their postmenopausal phase. Though it has been reported in few studies that the pharmacokinetics and pharmacodynamics characteristics of the drug inside a patient’s body is largely effects by inter individual variability, but presently, the drug is used for breast cancers of all configurations (Sanford and Plosker, 2008; USP, 2012). Anastrozole has been associated with a low rate of serum enzyme elevations during therapy and rare instances of clinically apparent liver injury (Shavi et al., 2017; Ekedahl et al., 2022). Like other anticancer drugs targeted and nanoformulations are being researched to overcome ANS, associated serious side effects due to uncontrolled delivery. Low solubility and short plasma half-life (FDA, 2011a; Pearce et al., 2012; Lam et al., 2016; Rahool et al., 2021).

##### 3.2.4.6 Exemestane

Aromatase works on the rate limiting stage of the estrogen biosynthesis process Exemestane (Aromasin) is a novel steroidal aromatase inhibitor (Untch and Jackisch, 2008) which was first approved for the treatment of postmenopausal breast cancer in Japan. Exemestane works on the principle of irreversibly binding with the pseudo-substrate with covalent bond so that it can inhibit the activity of aromatase enzyme It has been reported that the Exemestane has shown anti-tumor activity both conventional as well as testosterone treated- and ovariectomized postmenopausal models (Iirola et al., 2011; Li et al., 2013; Yavuz et al., 2007; Structures et al., 2023). Advanced research is focused on its safe and effective delivery through Novel approaches of drug delivery (Holmberg et al., 1997; Assesse Sophie, 2011; FDA, 2005a; FDA, 2015a; Accessdata, 1999).

##### 3.2.4.7 Goserelin

Goserelin, marketed in the name of Zoladex is a synthetic analogue of gonadotropin-releasing hormone (GnRH) (FDA, 2012d; FDA, 2020b; Gupta et al., 2021). which stimulates gonadotropin and sex hormone release in the short term, and then causes suppression with continued administration. It reduces the estrogen level in plasma/serum for pre- or perimenopausal women who is under Goserelin treatment. Goserelin is an effective alternative to surgery or estrogen therapy in prostate cancer palliation, and possibly to ovariectomy in premenopausal breast cancer (FDA, 2010c; FDA, 2018).

##### 3.2.4.8 MegestroAcetate

Megestrol acetate (MGA) is recognized as one of the first standard progestogen or progestational agent (Gregory et al., 1985; Ah-, 2018) effectively considered for advanced cancer (Schacter et al., 1989; Fjøsne et al., 2008) treatments because of its excellent safety profile. It is also considered as an effective treatment for anorexia-cachexia syndrome in cancer patients. It can penetrate the BBB when given in high doses. The response of this drug is considered comparable with tamoxifen, but megestrol acetate is more beneficial for patients who are suffering from cachexia. There is still a lot of research going on for considering this drug for the treatment of progesterone- and estrogen negative breast cancer (CDSCO, 2019; FDA, 2022).

##### 3.2.4.9 Methyl testosterone

Androgens are testosterone methyltestosterone, fluoxymesterone, and testolactone derivatives that are frequently used for palliative treatment of breast cancer in postmenopausal women who are receiving hormone therapy. The precise mechanism of androgens’ anticancer effect is unknown. However, it is assumed that androgens inhibit cell growth by preventing natural hormone transport into the cell (Vick and Hayton, 2001; Miles and White, 2018; Science direct, 2023a).

#### 3.2.5 Miscellaneous

##### 3.2.5.1 Everolimus

Everolimus is an oral Rapamycin (Natural Macrolide) derivative that selectively inhibits mTOR receptors. mTORC1 is a PI3K pathway signal transducer that becomes activated during human malignancies. Like Rapamycin, it has a binding interaction with FKBP12 and hinders the mTORC1 rather than mTORC2 complex formation. Everolimus has been reported to shunt tumor growth rate rather than promote cell death. It was approved in 2012 for use in postmenopausal women with HER2-negative, hormone-receptor-positive advanced breast cancer patients. It has been observed that Everolimus in advanced breast cancer patients gets quickly absorbed following oral administration, with a median time to peak blood levels. Bonizzi et al. (2019) reported Everolimus nano formulation increases drug responsiveness in resistant and low-responsive Breast Cancer Cell Lines (Bonizzi et al., 2019). Many research works claim that Co-delivery of Paclitaxel and Everolimus at the Optimal Synergistic Ratio may prove to be a Promising Solution for the Treatment of Breast Cancer (Houdaihed et al., 2018; Elena et al., 2019).

##### 3.2.5.2 Pamidronate

Osteolytic bone metastases commonly occur in patients with breast cancer (Evans et al., 2004). Pamidronate is nitrogen-containing bisphosphates used to treat bone metastases in breast cancer. Pamidronate inhibits bone resorption by adsorbing mineralized bone matrix on the surface of hydroxyapatite crystals. By impairing the attachment of osteoclast precursors to the mineralized matrix, pamidronate blocks their maturation into functioning osteoclasts, blocking osteoclast-mediated bone resorption that may lead to the weakening of the bone, fractures, and pain. It is available as an injection and lyophilized powder for reconstitution. Permitted inactive ingredients are mannitol and phosphoric acid. However, delivering drugs inside the diseased bone is a challenge. Nanomedicine which is able to target and deliver therapeutic agents to diseased bone sites could potentially provide an effective treatment option for different types of skeletal cancers. Yin et al. (84) demonstrated the use of pamidronate-functionalized nanoparticles of Polylactide to transport DOX to the bone microenvironment for the targeted treatment of OS. *In vivo* biodistribution of radiolabeled targeted Pam-NPs demonstrated enhanced bone tumor accumulation and prolonged retention compared with nontargeted NPs (Zhong et al., 2014; Yin and Tang, 2016). Pamidronate has been employed to target drugs inside the bone for the treatment of various other bone diseases. Numerous nano formulations containing Pamidronate as a targeting agent to bone are exploited. However, it is out of the scope of the contents of this paper. The drug may cause renal failure, embryogenic toxicity, electrolyte disorder, and osteonecrosis of the jaw.

##### 3.2.5.3 Gemcitabine (GEM)

GEM is a pyrimidine anti-metabolite that rapidly gets incorporated into DNA as a triphosphate. Gemcitabine (2, 2-difluoro-2-deoxycytidine, GEM) is a deoxycytidine analogue and is one of the most widely used anticancer drugs in the treatment of several types of solid tumours (Silvestris et al., 2008). In combination with paclitaxel, it is for first-line treatment of metastatic breast cancer after failure of prior anthracycline-containing adjuvant chemotherapy, unless anthracyclines were clinically contraindicated (Kushwah et al., 2018; Sung et al., 2021). The recommended dose for BC is 1250 mg/m2 over 30 min on Days 1 and 8 of each 21 days cycle. A study performed by Bijay et al. concludes that a combination of Gemcitabine and Iminoquid as nanoparticles has demonstrated better BC suppression by activation of the immune system. co-delivery of Gemcitabine in novel formulations has been experimented by many scientists (Mozar and Chowdhury, 2017; Kushwah et al., 2018; Lei et al., 2019; García-García et al., 2020; García-García et al., 2020). Various nanometer sized novel formulations carrying GEM individually are also reported (Kennedy, 1957; Mozar and Chowdhury, 2017), which claims to have the capability of improved delivery, lesser toxicity, and side effects.

##### 3.2.5.4 Fluoxymesterone

Fluoxymesterone is a synthetic androgenic anabolic steroid used in men and women to treat hypogonadism, delayed puberty, and breast neoplasms. Fluoxymesterone, has shown to be effective in postmenopausal women with advanced breast cancer (Goldhirsch et al., 1982a; Turner et al., 2023). Its side effects are mostly those associated with the physiologic effects of male hormone, such as virilization with frontal baldness, plethora and acne, hirsutism, fluid retention, and, less frequently, increased libido and clitoral hypertrophy (Kennedy, 1958; Goldhirsch et al., 1982). One case report describes an unusual case of ataxia and unsteadiness of gait caused by Fluoxymesterone therapy (Kennedy, 1958; Science direct, 2023b). It is marketed as oral pill of 10 mg under the brand names Halotestin and Ultandren.

##### 3.2.5.5 Ixabepilone

Ixabepilone is an epothilone analog developed by Bristol-Myers Squibb (FDA, 2005b). It is considered as a new member of anti-neoplastic drugs from 2007 after being approved by US-FDA (Egerton, 2008; Ibrahim, 2021). Ixabepilone is a micro-tubule stabilizer approved as a monotherapy and in combination with Capecitabine for the treatment of metastatic breast cancer in patients with demonstrated resistance to Anthracyclines and Taxanes. Ixabepilone was specially derived for patients who have developed resistance with some other therapies (Huang et al., 2010; Thomas et al., 2015), (Thomas et al., 2022). Epothilones have higher affinity for β-tubulin and are not P-gp substrates. Certain smedications, including (but not limited to) Verapamil, Ketoconazole, Rifampin, Phenytoin, and Phenobarbital, can interfere with this medication (Chuang et al., 2010). CYP3A4 inducers and inhibitors may cause decrease and increase plasma concentration, hence the dose needs to be adjusted accordingly for the patients (Bagegni et al., 2022). It is administered through injection and branded as Ixempra. A pre-formulated liposomal version of Ixabepilone has been reported to have optimal *in vivo* performance (Hortobagyi, 2018).

##### 3.2.5.6 Pembrolizumab

Pembrolizumab is a humanized anti-programmed cell death monoclonal IgG4 kappa anti-PD1 antibody. Binding of Pembrolizumab to PD1 does not engage FC receptors which is a surface protein mainly found on the surface of cells like B-lymphocytes, natural killer cells, etc. FC receptors plays a significant role in activating immune complexes for healthy cell development. The 50% effective inhibitory concentration in T-cell activation assays ranges from 0.1 to 0.3 nm. This drug has been reported to be used for triple negative breast cancer patients who has active autoimmune disease (March 2017). It is sold in market as injection formulations of 50 mg single dose vial as powder for reconstitution and 25 mg/mL single dose vial as solution (Hortobagyi, 2018; Bagegni et al., 2022).

##### 3.2.5.7 Ribociclib

Ribociclib (RIB) is an oral cyclin-dependent kinase 4 and 6 (CDK4/6) inhibitor that has been recommended in year 2017 as a preferred regimen for the treatment of premenopausal women with HR-positive, HER2-negative Breast cancer. It has been reported to inhibit the phosphorylation of retinoblastoma protein which further arrests cell cycle progression in G1 phase (FDA, 2017; Slamon et al., 2021), (Ahmed and Fatima, 2022; SartaI, 2022). The recommended starting dose of Ribociclib is 600 mg orally (three 200 mg tablets) taken once daily with or without food for 21 consecutive days followed by 7 days off treatment. Ribociclib Nanostructured Lipid Carrier are reported to have overcome the inherent lacuna of limited bioavailability (Fei and Yoosefian, 2021). Ribociclib-Loaded Ethylcellulose-Based Nanosponges Formulation reportedly demonstrated better Cytotoxic Potential against Breast Cancer (Swaminathan et al., 2016; Ji et al., 2020). Ribociclib loaded Polymeric micelles showed anti-cancer potential at much lower doses of Ribociclib (Ji et al., 2020; Robson et al., 2017). s

##### 3.2.5.8 Olaparib

Olaparib represents a rational strong class of drugs called PARP (Poly ADP-ribose polymerase) inhibitors administered orally for metastatic and germline BRCA mutation in breast cancer. Clinical potential of Olaparib monotherapy has been documented in somatic or germline BRCA1/2 metastatic type of breast cancer therapy (Robson et al., 2017; Kuemmel et al., 2020), (Caulfield et al., 2019; Gote et al., 2021). Olaparib is the first treatment approved specifically for BRCA mutation carriers with HER2-negative metastatic breast cancer and previous treatment with chemotherapy in the neoadjuvant, adjuvant, or metastatic setting (Kuemmel et al., 2020; Structures et al., 2023c). It has been reported to enhance chemotherapy without increasing toxicity when administered in nanoparticle form (Migotto et al., 2018). Many novel formulations have been experimented by different independent research groups to enhance oral efficacy, reduced tumor proliferation, to inhibit growth and metastasis (Cortesi et al., 2021).

##### 3.2.5.9 Tolazoparib tasylate

Talazoparib was approved by the FDA for use in germline BRCA mutated, HER2 negative, locally advanced or metastatic breast cancer on. 16 October 2018 (Zhang et al., 2019). It is a poly ADP ribose polymerase (PARP) inhibitor, prevents PARP-mediated DNA repair this enhances the accumulation of DNA stand breaks, promoting genomic instability eventually leading to apoptosis (Zhang et al., 2019). This drug has been recommended for the use for only those patients who have already undergone an ineffective hormonal therapy for breast tumor. Presently, Tolazoparib is available as a capsule with the brand name Talzenna developed by Pfizer.

## 4 Discussion and conclusion

Drug discovery for Breast Cancer has always been an area of interest and important priority for researchers as even today there is no drug/drug combination which can promise 100% side effects/adverse effects free treatment of Breast Cancer, the most common malignancy in women across the world. In all of 206 anticancer drugs approved by United States-FDA, 39 are for breast cancer treatment alone.

Though the first drug for breast cancer was approved in 1953 the initial research was observed to be quite slow as only 8 drugs could get approval till 1988 for breast cancer indication. However, in later decades the research acquired momentum and offered various treatment choices (Pietrangelo, 2021). Initially several breast cancer approvals granted by FDA were dominated by Cytotoxic drugs with Methoterxate being the first and most widely used. Later in 1970s hormonal drugs, specially Estrogen Receptors Modulators, i.e., Tamixifen eclipsed the market/limelight and marked the inception of precision medicine in cancer. Another milestone in the history of breast cancer therapy was the approval of Trastuzumab, a monoclonal antibody that targets human epidermal growth factor receptor 2 (HER2). Among these hormonal therapy drugs, i.e., aromatase inhibitor (anastrozole, letrozole, or exemestane) in combination with a CDK 4/6 inhibitor, anti-estrogens (fulvestrant and tamoxifen), and Targrted therapy, i.e., Trastuzumab, Pertuzumab, ado-Trastuzumab Emtansine, Trastuzumab Deruxtecan, Tucatinib, Neratinib, Lapatinib, are used as first line treatment for ER/PR positive and HER positive breast cancer respectively. However, the chemotherapy is left as first choice of treatment if the patient is the victim of TNBC (Triple Negative Breast Cancer) (National Cancer Institute, 2017). Generally, these are employed as combination therapy. Most commonly employed combinations are AC (Adriamycin and Cyclophosphamide), AC-T (Adriamycin, Cyclophosphamide and Taxol-Peclitexal), CAF (Cyclophosphamide, Adriamycin and 5-Flurouracil), CMF (Cyclophosphamide, Methotrexate and 5-Flurouracil), FEC (5-Flurouracil, Epirubici Hydrochloride and Cyclophosphamide) and, TAC (Taxol, Adriamycin and Cyclophosphamide) (Álvarez, 2010).

Drugs used to treat breast cancer are considered systemic therapies because they can reach cancer cells almost anywhere in the body. Some can be administered orally, through intramuscular route, or as an intravenous injection or infusion. Depending on the type of breast cancer, different types of drug treatment might be used. In this article we have compiled information about mechanism of action, approval status, limitations and side effects, available commercial formulations, novel and targeted drug delivery systems which have been researched for various types of breast cancer and the formulations which are under clinical trial. This information may considerably help a formulation scientist in deciding the drug on which advanced research is required/possible in terms of formulation development. An idea of side effects and adverse effects of each drug shall further help the scientist for gaining information in designing a drug or a delivery system from a single manuscript which may reduce or eliminate these side effects by targeting to desired sites or by preventing the drug to reach at sites where side effects are observed. Moreover, the information also saves the scientists from the efforts which may be wasted by duplication of research.

A formulator may further decide to work on dose reduction and co-delivery of drugs in single formulation with the acquaintance of frequently used drug combinations in treatments which will surely enhance patient compliance and adherence to the treatment. This article encompasses all that information which may help a scientist to refer to for his further research in the field of breast cancer.

Optimal outcomes for breast cancer therapy are immensely dependent on timely diagnosis followed by effective multidisciplinary approach of cancer treatment. The history of cancer in the medical background started long back in ancient Greek and Egyptian civilizations. There have been a lot of discoveries over the centuries that have helped in the evolution of the therapeutic approach towards breast cancer treatment. The most important fundamental breakthrough in medical oncology took place in the beginning of ’80s when the immergence of specific drugs came intended for molecular targets came into existence. All the chemotherapeutic as well as targeted drugs have improvised the survival and living quality of breast cancer patients (Zardavas and Piccart-Gebhart, 2016; Jerusalem et al., 2018; Waks and Winer, 2019; Junnuthula et al., 2022). The advancement in drug discovery has also led to the further enhancement of clinical oncology with an introduction of monoclonal antibodies into the treatment regime. Likewise, introduction of new drugs for better management will always remain continuous process where it has already been reported that various novel biotechnological drugs have depicted promising preclinical results. Table 1 contains the chronological list of all the drugs till date along with their year of approval, brand name and their formulations which are available in the market, or under clinical trial respectively. Moreover, the novel formulations are getting humungous attentions of researchers and maufacturers as these can provide targeted treatment which is devoid of side/toxic effects and improve quality of life. Table 2 summerizes the novel formulations which are under clinical trials and are promising in better management of breast cancer. Graphical presentation of the data is depicted in Figure 6. Therefore, to conclude, this exhaustive review article will become a one-stop solution for breast oncology scientist and medical professionals to refer to. This aims to reduce to hassle of data searching and enhance the ease of data access about the approved breast cancer drugs for further research.

**TABLE 1:** A Comprehensive Account of Commercial Formulations of Drugs Approved for first line, neoadjuvant, and Adjuvant therapy of various types of Breast Cancers by USFDA [Approval Year wise General information 1953 to 2023].

# S.No.	Drug name	Year of approval	Number of Formul-ations in market	Dosage forms in market	Notable formulations	Manufacturers	Recommended dose	Condition of breast cancer	Drug delivery type	Ref
1	Methotrexate	1953	48	i. Injection	i. Biotrexate ii. Auratrex iii. Trexjoy	i. Biochem	i. 15 mg/mL)	Primary metastatic breast cancer	Combination	Accessdata, (2019)
				ii. Tablet		ii. AHPL	ii. 2.5 mg			
				iii. Topical gel		ii. Intas	ii. 1%w/w *20g			
2	Fluoxymesterone	1956	2	i. Tablet	i. Androxy ii. Halotestin	i. Upsher-Smith	i. 10–40 mg daily	Androgen-responsive, advanced, metastatic (skeletal) breast cancer	Single	FDA, (1959)
				ii. Tablet						
3	Tepadina	1957	4	i. Injection (iv)	i. Thiotepa	Adiennesa	0.3-0.4 mg/kg	First line andsecond line breast cancer	Combination	USP, (2012)
4	Cyclophosphamide	1959	25	i. Injection (iv)	i. Uniphes ii. Uniphos	i. United biotech	i. 200mg	Retinoblastoma breast carcinoma	Single and combination both	FDA (2012c); FDA (2013)
				ii. Tablet		ii. United biotech	ii. 50 mg tablet.			
5	Vinblastin Sulfate	1965	4	i. Injection (iv)	i. Chemoblast ii. Vblastin	i. Neon lab	i. 10 mg/mL	Neoadjuvant, adjuvant or in first line metastatic breast cancer	Combination	FDA (2010a); Lam et al. (2016)
				ii. Injection (iv)		ii. Chandra bhagat pharma	ii. 10 mg/mL			
6	5-Fulurouracil	1970	7	i. capsule	i. Lupral ii. Tegafi	i. Lupin	i. 10mg	Adenocarcinoma of the breast cancer	Single	Al-Batran et al. (2016); Rahool et al. (2021)
				ii. Tablet		ii. Intas	ii. 14mg			
				iii. Injection (iv)		iii. Spectrum pharmaceuticals Inc	iii. 600 mg/m^2^ intravenously			
7	Methyl testosterone	1973	2	i. Injection ii. capsule	i. Mixagen	i. Organon ii. Valeant Pharmaceuticals	i. 1mL ii. 10–50 mg	Breast Carcinoma andPostpartum Breast Painand Engorgement in females	Single and combination both	Ixabepilone, (2006)
8	Doxorubicin	1974	58	i. Injection (iv)	i. Dobicin ii. Dobixin	i. Chandra bhagat pharma	i. 10mg/5 mL	Metastatic breast cancer	Single	FDA (2012a); Pearce et al. (2012)
				ii. Injection (iv)		ii. Zydus pharma	ii. 2 mg/mL			
9	Tamoxifen	1977	21	i. Tablet	i. Blastofen ii. Valodex	i. Chandra bhagat pharma	i. 10mg per day	Estrogen receptor-positive metastatic breast cancer	Single	USP (2012); Wang et al. (2018)
				ii. Tablet		ii. Samarth pharma				
10	Goserelin Acetate	1989	3	i. Injection (iv)	i. Goselin ii. Zoladex-LA	i. Bharat serum	i. 3.6 mg	Advanced breast cancer in pre- and perimenopausal women	Single and combination both	FDA, (2011)
				ii. Injection (iv)		ii. AstraZeneca pharmaceutical LP	ii. 6.8 mg			
11	Paclitaxel	1992	7	i. Injection (iv)	i. Abraxane ii. Adpaxil	i. Biochem	i. 20 mL	First line treatment for triple negative breast cancer	Single and combination both	FDA (1995); FDA (2011)
				ii. Injection (iv)		ii. Adley	ii. 30mL			
12	Anastrozole	1995	34	i. soft capsule	i. Adove ii. Altraz	i. Akumemtis	i. 1 mg capsule once daily	Used for first line breast cancer treatment	Single	Iirola et al. (2011)
				ii. Tablet		ii. Alkem	ii. 1 mg tablet once daily			
13	Pamidronate	1996	1	Injection	Aredia	Novartis Pharmaceuticals Corp	30 mg or 90 mg intravenously	Osteolytic Bone Metastases of Breast Cancer	Single	Holmberg et al. (1997); FDA (2009)
14	Gemcitabine	1996	40	i. Injection (iv)	i. Abingem ii. Biogem	i. Miracalus	i. 200mg	First line treatment of metastatic breast cancer	Single and combination both	Lei et al. (2019)
				ii. Injection (iv)		ii. United biotech	ii. 200mg			
15	Docetaxel	1996	56	i. Injection (iv)	i. Apidry ii. Docax	i. Taj pharma	i. 20mg	Locally advance or Metastaic breast cancer	Single and combination both	Luo et al. (2020b)
				ii. Injection (iv)		ii. Neon labs	ii. 20mg			
16	Letrozole	1997	56	i. Tablet	i. Anolet ii. Arohin	i. Svizera	i. 2.5 mg once daily	First line& Second line or Advance treatment of breast cancer	Single	Assesse Sophie, (2011)
				ii. Tablet		ii. Grace	ii. 2.5 mg once daily			
17	Toremifene	1997	1	Tablet	i. Fareston	GTx Inc	60 mg once daily	Estrogen receptor-positive metastatic	Single	FDA (2005a); Assesse Sophie (2011)
								Breast cancer		
18	Megestral Acetate	1998	6	i. Tablet	i. Megahenz ii. Megasty	i. Alniche	i. 40 mg	Hormonal therapy advanced breast cancer	Single and combination both	FDA, (2015a)
				ii. Tablet		ii. Alniche	ii. 160mg			
19	Transtuzumab	1998	18	i. Injection (iv)	i. Herceptin ii. Biceltis	i. Roche pharma ii. Genentech Inc	i. 440mg ii. 600mg	HER-2 overexpressing breast cancer	Single	Schlotter et al. (2008)
20	Capecitabine	1998	33	i. Tablet ii. Tablet	i. Cacit ii. Capcel	i. Biochem ii. Celon pharma	i. 500 m tablet twice daily ii. 500 m tablet twice daily	Metastaic breast cancer	Single and combination both	Accessdata, (1999)
21	Exemestane	1999	7	i. Tablet ii. Tablet	i. Aromasin ii. Exeget	i. Pfizer Pharma ii. Getwell	i. 25 mg tablet once daily i. 25 mg tablet once daily	Advance treatment of breast cancer	Single	FDA, (2020b)
22	Epirubicin	1999	33	i. Injection (iv) ii. Injection (iv)	i. 4-EPPEDO 10 ii. Biorubin	i. Miracalus ii. Biochem	i. 2–10 mg/m^2^ daily ii. 2–10 mg/m^2^ daily	Primary breast cancer	Single	FDA, (2010c)
23	Fulvestrant	2002	1	injection (im)	i. Faslodex	AstraZeneca United Kingdom Limited	5 mL	HR positive Metastatic breast cancer	Single	Nathan and Schmid (2017); FDA (2018)
24	Ixabepilone	2007	1	injection (iv)	i. Ixempra	Bristol Mayers	15 mg	Locally advanced or metastatic breast cancer	Single and combination both	Chuang et al. (2010); Ibrahim (2021)
25	Lapatinib	2007	8	i. Tablet	i. Combinib ii. Herduo	i. Cipla	i. 250mg	Advanced or metastatic breast cancer	Combination	FDA, (2012b)
				ii. Tablet		ii. Natco	ii. 250mg			
26	Everolimus	2009	14	i. Tablet	i. Afinitor ii. Advacam	i. Novartis pharmaceutical	i. 5mg once daily	Advance HER-2 negative Breast cancer	Single and combination both	Zhang et al. (2003); FDA (2010b)
				ii. Tablet		ii. Biochem	ii. 0.25 mg			
27	Eribulin mesylate	2010	5	i. Injection (iv)	i. Brutravon ii. Emcure	i. Taj pharma	i. 1.5 mg/m 2	First line metastatic breast cancer	Single	Mcbride and Butler, (2012)
				ii. Injection (iv)		ii. Embremma	ii. 0.5 mg/m 2			
28	Pertuzumab	2012	1	injection (iv)	i. Perjeta	Genentech Inc	420 mg/14 mL	HER2-positive metastatic breast cancer	Single and combination both	FDA, (2015b)
29	Palbociclib	2015	1	Capsule	i. Ibrance)	Pfizer Pharmacia &Upjohn Co	125 mg	HER2-negative advanced or metastatic breast cancer	Combination	FDA, (2022)
30	Abemaciclib	2017	1	Tablet	i. Verzenio	Eli lily (European medical Agency)	150 mg	First line metastatic breast cancer	Combination	CDSCO (2019); Voli et al. (2020)
31	Ribociclib	2017	1	Tablet	i. Kryxana	Novartis pharm	200 mg orally	HER-2 Negative advanced or metastatic breast cancer	Combination	Miles and White, (2018)
32	Neratinib Maleate	2017	1	Tablet	i. Nerlynx	Pierre Fabre medicament production	240 mg	HER-2 positive metastatic breast cancer	Single	FDA (2017b); Zhang et al. (2020)
33	Olaparib	2018	1	Tablet	i. Lynparza	AstraZeneca United Kingdom Limited	150 mg	HER-2 Negative breast cancer	Single and combination both	Caulfield et al. (2019); Zhang et al. (2019)
34	Tolazoparib	2018	1	Capsule	i. Talzenna	Pfizer Lab	1 mg	HER-2 Negative locally advanced or metastatic breast cancer	Single	FDA (2021a); Fan (2021)
35	Atezolizumab	2019	1	i. Injection (iv)	i. Tecentriq	i. Roche pharm	i. 840 mg/mL	Triple negative breast cancer	B Single and combination both	FDA (2019); FDA (2020a)
36	Alpelisib	2019	15	i. Injection (iv)	i. Aredia ii. Arimidex	i. Novartis (FDA Approved)	i. 90mg per day	HER-2 Negative advanced or metastatic breast cancer	Single and combination both	Egerton (2008); Armaghani and Han (2020)
				ii. Tablet		ii. AstraZeneca	i. 1 mg			
37	Pembrolizumab	2020	1	injection (iv)	i. Keytruda	Merck and Co Inc	100 mg	Metastatic triple negative breast cancer	Single and combination both	Karthick and Panda (2019); FDA (2021b)
38	Margetuximab	2020	1	injection (iv)	i. Margenza	MacroGenics	15 mg/kg over 120 min	HER-2 positive metastatic breast cancer	Single and combination both	Goss and Tye (1997); Gavas et al. (2021)
39	Tucatinib	2020	1	Tablet	i. Tukysa	Seatle genetics	300 mg	HER-2 positive advanced or metastatic breast cancer	Combination	Elith et al. (2006); FDA (2023)

**TABLE 2:** List of Various novel formulations in clinical trial for various conditions of breast cancer.

# S.No.	Drug	Type of novel formulation	Condition of breast cancer	NCT number	Clinical trial phase	References
1	Cyclophosphamide (Cytoxon)	Nanoparticles	Retinoblastoma breast carcinoma	NCT00629499P	Phase II	Cho and Moniri (2016); Banu et al. (2014)., Clinical Trial.gov
2	Doxorubicin Hydrochloride (Rubex)	Liposome	Triple negative breast cancer	NCT03164993	Phase II	Wissner and Mansour (2008); Wong et al. (2006); O’Brien et al. (2004)., Clinical Trial.gov
		Nanoparticles		NCT03606967	Phase II	
3	Tamoxifen (Soltamox)	Nanoparticles	Metastatic Breast Cancer	NCT04997941	Phase II	Dreaden et al. (2009); Maji et al. (2014); Zhang et al. (2019)., Clinical Trial.gov
4	Gemcitabine Hydrochloride (Gemzar)	Nanoparticles	First line breast cancer treatment	NCT00662129	Phase II	De Angel et al. (2013); Devi et al. (2020); Sung et al. (2021)., Clinical Trial.gov
5	Docetaxel (Taxotere)	Nanosomal lipid suspension	First line treatment of metastatic breast cancer	NCT03671044	Phase III	Sahu et al. (2016); Falvo et al. (2021)., Clinical Trial.gov
6	Transtuzumab (Herceptin)	Injectable solution	First line& Second line or Advance treatment of breast cancer	NCT01875367	Phase III	Lo et al. (2019); Yassemi et al. (2020)., Clinical Trial.gov
7	Ellence (Epirubin)	Nanoparticles	HR positive Metastatic breast cancer	NCT00110695	Phase II	Chang et al. (2009); Lan et al. (2018)., Clinical Trial.gov
8	Abraxane (Nab-Paclitaxel)	Nanoparticles for injectable suspension	First line treatment for triple negative breast cancer	NCT00251472	Phase II	Lan et al. (2018)., Clinical Trial.gov
9	Paclitaxel (Taxol)	Nanoparticles	Locally advanced or MSBC	NCT00629499	Phase II	FDA (1995); Yang et al. (2009); Rasaneh and Zahabi (2016)., Clinical Trial.gov
			Advanced or MBC			
			Advance HER-2 negative Breast cancer			

**FIGURE 6:**
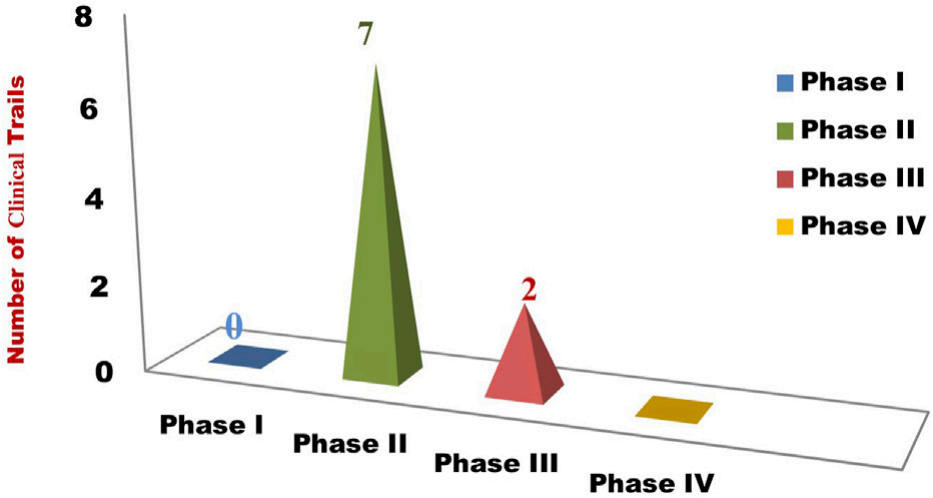
Under trial (Clinical) **N**ovel formulations for breast cancer.
