# Supplementary material for: A review of FDA approved drugs and their formulations for the treatment of breast cancer
Source: Front Pharmacol. 2023 Jul 28;14:1184472. doi: 10.3389/fphar.2023.1184472 (PMC10416257; doi:10.3389/fphar.2023.1184472)
Supplement: Supplementary file 1 [file Table1.DOCX]

**Table 1**: A Comprehensive Account of Commercial Formulations of Drugs Approved for first line, neoadjuvant, and Adjuvant therapy of various types of Breast Cancers by USFDA [Approval Year wise General information 1953 to 2023].

| # | Drug Name | Year of  Approval | Number of Formul-ations in market | Dosage forms in Market | Notable formulations | Manufacturers | Recommended Dose | Condition of Breast Cancer | Drug Delivery Type | Ref. |
| --- | --- | --- | --- | --- | --- | --- | --- | --- | --- | --- |
| 1 | Methotrexate | 1953 | 48 | i. injection  ii. tablet  iii. topical gel | i. Biotrexate ii. Auratrex iii. Trexjoy | i. Biochem  ii. AHPL  ii. Intas | i. 15mg/mL)  ii. 2.5 mg  ii. 1%w/w *20g | Primary metastatic breast cancer | Combination | [73] |
| 2 | Fluoxymesterone | 1956 | 2 | i. tablet  ii. tablet | i. Androxy ii. Halotestin | i. Upsher-Smith | i. 10–40 mg daily | Androgen-responsive, advanced, metastatic (skeletal) breast cancer | Single | [216] |
| 3 | Tepadina | 1957 | 4 | i. injection (iv) | i. Thiotepa | Adiennesa | 0.3-0.4 mg/kg | First line &second line breast cancer | Combination | [217] |
| 4 | Cyclophosphamide | 1959 | 25 | i. injection (iv)  ii. tablet | i. Uniphes ii. Uniphos | i. United biotech  ii. United biotech | i. 200mg  ii. 50 mg tablet. | Retinoblastoma breast carcinoma | Single and combination both | [62,299] |
| 5 | Vinblastin Sulfate | 1965 | 4 | i. injection (iv)  ii. injection (iv) | i. Chemoblast ii. vblastin | i. Neon lab  ii. Chandra bhagat pharma | i. 10mg/mL  ii. 10mg/mL | Neoadjuvant, adjuvant or in first line metastatic breast cancer | Combination | [218,297] |
| 6 | 5-Fulurouracil | 1970 | 7 | i. capsule  ii. tablet  iii. injection (iv) | i. Lupral ii. Tegafi | i. Lupin  ii. Intas  iii. Spectrum pharmaceuticals Inc. | i. 10mg  ii. 14mg  iii. 600 mg/m^2^ intravenously. | Adenocarcinoma of the breast cancer | Single | [219,298] |
| 7 | Methyl testosterone | 1973 | 2 | i. injection  ii. capsule | i. Mixagen | i. Organon  ii. Valeant Pharmaceuticals | i. 1mL  ii. 10 to 50 mg | Breast Carcinoma and [Postpartum](https://www.rxlist.com/postpartum/definition.htm) [Breast Pain](https://www.rxlist.com/breast_pain/definition.htm) and Engorgement in females | Single and combination both | [190] |
| 8 | Doxorubicin | 1974 | 58 | i. injection (iv)  ii. injection (iv) | i. Dobicin ii. Dobixin | i. Chandra bhagat pharma  ii. Zydus pharma | i. 10mg/5mL  ii. 2 mg/mL | Metastatic breast cancer | Single | [220,209] |
| 9 | Tamoxifen | 1977 | 21 | i. tablet  ii. tablet | i. Blastofen ii. Valodex | i. Chandra bhagat pharma  ii. Samarth pharma | i. 10mg per day. | Estrogen receptor-positive metastatic breast cancer | Single | [217,150] |
| 10 | Goserelin Acetate | 1989 | 3s | i. injection (iv)  ii. injection (iv) | i. Goselin ii. Zoladex-LA | i. Bharat serum  ii. AstraZeneca pharmaceutical LP | i. 3.6 mg  ii. 6.8mg | Advanced breast cancer in pre- and perimenopausal women | Single and combination both | [221] |
| 11 | Paclitaxel | 1992 | 7 | i. injection (iv)  ii. injection (iv) | i. Abraxane ii. Adpaxil | i. Biochem  ii. Adley | i. 20 mL  ii. 30mL | First line treatment for triple negative breast cancer | Single and combination both | [222,300] |
| 12 | Anastrozole | 1995 | 34 | i. soft capsule  ii. tablet | i. Adove ii. Altraz | i. Akumemtis  ii. Alkem | i. 1 mg capsule once daily.  ii. 1 mg tablet once daily | Used for first line breast cancer treatment | Single | [223] |
| 13 | Pamidronate | 1996 | 1 | Injection | Aredia | Novartis Pharmaceuticals Corp. | 30mg or 90mg intravenously. | Osteolytic Bone Metastases of Breast Cancer | Single | [224,301] |
| 14 | Gemcitabine | 1996 | 40 | i. injection (iv)  ii. injection (iv) | i. Abingem ii. Biogem | i. Miracalus  ii. United biotech | i. 200mg  ii. 200mg | First line treatment of metastatic breast cancer | Single and combination both | [183] |
| 15 | Docetaxel | 1996 | 56 | i. injection (iv)  ii. injection (iv) | i. Apidry ii. Docax | i. Taj pharma  ii. Neon labs | i. 20mg  ii. 20mg | Locally advance or Metastaic breast cancer | Single and combination both | [103] |
| 16 | Letrozole | 1997 | 56 | i. tablet  ii. tablet | i. Anolet ii. Arohin | i. Svizera  ii. Grace | i. 2.5 mg once daily  ii. 2.5 mg once daily | First line& Second line or Advance treatment of breast cancer | Single | [225] |
| 17 | Toremifene | 1997 | 1 | Tablet | i. Fareston | GTx Inc. | 60 mg once daily | Estrogen receptor-positive metastatic  Breast cancer | Single | [226,303] |
| 18 | Megestral Acetate | 1998 | 6 | i. tablet  ii. tablet | i. Megahenz ii. megasty | i. Alniche  ii. Alniche | i. 40 mg  ii. 160mg | Hormonal therapy advanced breast cancer | Single and combination both | [227] |
| 19 | Transtuzumab | 1998 | 18 | i. injection (iv) | i. Herceptin ii. Biceltis | i. Roche pharma  ii. Genentech Inc. | i. 440mg  ii. 600mg | HER-2 overexpressing breast cancer | Single | [128] |
| 20 | Capecitabine | 1998 | 33 | i. tablet  ii. tablet | i. Cacit ii. Capcel | i. Biochem  ii. Celon pharma | i. 500 m tablet twice daily ii. 500 m tablet twice daily. | Metastaic breast cancer | Single and combination both | [228] |
| 21 | Exemestane | 1999 | 7 | i. tablet  ii. tablet | i. Aromasin ii. Exeget | i. Pfizer Pharma  ii. Getwell | i. 25 mg tablet once daily  i. 25 mg tablet once daily | Advance treatment of breast cancer | Single | [229] |
| 22 | Epirubicin | 1999 | 33 | i. injection (iv)  ii. injection (iv) | i. 4-EPPEDO 10 ii. Biorubin | i. Miracalus  ii. Biochem | i. 2-10mg/m^2^ daily  ii. 2-10mg/m^2^ daily | Primary breast cancer | Single | [230] |
| 23 | Fulvestrant: | 2002 | 1 | injection (im) | i. Faslodex | AstraZeneca UK Limited | 5mL | HR positive Metastatic breast cancer | Single | [231,242] |
| 24 | Ixabepilone | 2007 | 1 | injection (iv) | i. Ixempra | Bristol Mayers | 15mg | Locally advanced or metastatic breast cancer | Single and combination both | [196,192] |
| 25 | Lapatinib | 2007 | 8 | i. tablet  ii. tablet | i. Combinib ii. Herduo | i. Cipla  ii. Natco | i. 250mg  ii. 250mg | Advanced or metastatic breast cancer | Combination | [232] |
| 26 | Everolimus | 2009 | 14 | i. tablet  ii. tablet | i. Afinitor ii. Advacam | i. Novartis pharmaceutical  ii. Biochem | i. 5mg once daily  ii. 0.25 mg | Advance HER-2 negative Breast cancer | Single and combination both | [63,304] |
| 27 | Eribulin mesylate | 2010 | 5 | i. injection (iv)  ii. injection (iv) | i. Brutravon ii. Emcure | i. Taj pharma  ii. Embremma | i. 1.5 mg/m 2  ii. 0.5 mg/m 2 | First line metastatic breast cancer | Single | [115] |
| 28 | Pertuzumab | 2012 | 1 | injection (iv) | i. Perjeta | Genentech Inc. | 420 mg/14mL | HER2-positive metastatic breast cancer | Single and combination both | [233] |
| 29 | Palbociclib | 2015 | 1 | Capsule | i. Ibrance) | Pfizer Pharmacia &Upjohn Co | 125mg | HER2-negative advanced or metastatic breast cancer | Combination | [234] |
| 30 | Abemaciclib | 2017 | 1 | Tablet | i. Verzenio | Eli lily (European medical Agency) | 150mg | First line metastatic breast cancer | Combination | [235,141] |
| 31 | Ribociclib | 2017 | 1 | Tablet | i. kryxana | Novartis pharm | 200mg orally | HER-2 Negative advanced or metastatic breast cancer | Combination | [236] |
| 32 | Neratinib  Maleate | 2017 | 1 | Tablet | i. Nerlynx | Pierre Fabre medicament production | 240mg | HER-2 positive metastatic breast cancer | Single | [237,312] |
| 33 | Olaparib | 2018 | 1 | Tablet | i. Lynparza | AstraZeneca UK Limited | 150 mg | HER-2 Negative breast cancer | Single and combination both | [238,207] |
| 34 | Tolazoparib | 2018 | 1 | Capsule | i. Talzenna | Pfizer Lab | 1 mg | HER-2 Negative locally advanced or metastatic breast cancer | Single | [239,305] |
| 35 | Atezolizumab | 2019 | 1 | i. injection (iv) | i. Tecentriq | i. Roche pharm | i. 840 mg/mL | Triple negative breast cancer | B Single and combination both | [240,133] |
| 36 | Alpelisib | 2019 | 15 | i. injection (iv)  ii. tablet | i. Aredia ii. Arimidex | i. Novartis (FDA Approved)  ii. AstraZeneca | i. 90mg per day  i. 1 mg | HER-2 Negative advanced or metastatic breast cancer | Single and combination both | [191,138]. |
| 37 | Pembrolizumab | 2020 | 1 | injection (iv) | i. Keytruda | Merck & Co Inc. | 100 mg | Metastatic triple negative breast cancer | Single and combination both | [316,311]. |
| 38 | Margetuximab | 2020 | 1 | injection (iv) | i. Margenza | MacroGenics | 15mg/kg over 120 minutes | HER-2 positive metastatic breast cancer | Single and combination both | [164,310]. |
| 39 | Tucatinib | 2020 | 1 | Tablet | ( i)Tukysa | Seatle genetics | 300mg | HER-2 positive advanced or metastatic breast cancer | Combination | [182,307]. |

Table 2. List of Various novel formulations in licnical trial for various conditions of breast cancer

| **#** | **Drug** | **Type of novel formulation** | **Condition of Breast cancer** | **NCT Number** | **Clinical Trial Phase** | **References** |
| --- | --- | --- | --- | --- | --- | --- |
| **1** | **Cyclophosphamide**  **(**Cytoxon) | Nanoparticles | Retinoblastoma breast carcinoma | NCT00629499P | Phase II | [55,322]., Clinical Trial.gov |
| **2** | **Doxorubicin Hydrochloride**  **(**Rubex) | Liposome | Triple negative breast cancer | NCT03164993 | Phase II | [145,331,332]., Clinical Trial.gov |
|  |  | Nanoparticles |  | NCT03606967 | Phase II |  |
| **3** | **Tamoxifen**  (Soltamox) | Nanoparticles | Metastatic Breast Cancer | NCT04997941 | Phase II | [238,320,321]., Clinical Trial.gov |
| **4** | **Gemcitabine Hydrochloride** (Gemzar) | Nanoparticles | First line breast cancer treatment | NCT00662129 | Phase II | [267,329,330 ]., Clinical Trial.gov |
| **5** | **Docetaxel** (Taxotere) | Nanosomal lipid suspension | First line treatment of metastatic breast cancer | NCT03671044 | Phase III | [99,306]., Clinical Trial.gov |
| **6** | **Transtuzumab** (Herceptin) | Injectable solution | First line& Second line or Advance treatment of breast cancer | NCT01875367 | Phase III | [161,328]., Clinical Trial.gov |
| **7** | **Ellence**  (Epirubin) | Nanoparticles | HR positive Metastatic breast cancer | NCT00110695 | Phase II | [153,326]., Clinical Trial.gov |
| **8** | **Abraxane**  (Nab-Paclitaxel) | Nanoparticles for injectable suspension | First line treatment for triple negative breast cancer | NCT00251472 | Phase II | [153]., Clinical Trial.gov |
| **9** | **Paclitaxel**  (Taxol) | Nanoparticles | Locally advanced or MSBC  Advanced or MBC  Advance HER-2 negative Breast cancer | NCT00629499 | Phase II | [222,324,325]**.,** Clinical Trial.gov |
